# Supplementary material for: Do I Belong Here? Confronting Imposter Syndrome at an Individual, Peer, and Institutional Level in Health Professionals
Source: MedEdPORTAL. 2021 Jul 6;17:11166. doi: 10.15766/mep_2374-8265.11166 (PMC8257750; doi:10.15766/mep_2374-8265.11166)
Supplement: Supplementary file 1 — Facilitator Guide.docxWorkshop Handout.docxFacilitator Lesson Plan.docxPowerPoint Slides.pptxWorkshop Evaluation Form.docx [file mep_2374-8265.11166-s001.zip › A. Facilitator Guide.docx]

**Title: Do I Belong Here? A Workshop to Promote Diversity and Cultivate Success in Health Professionals Experiencing Imposter Syndrome.**

**Authors:** Nancy Rivera, MD, MS, Elana A. Feldman, MD, Dimitri A. Augustin, MD, Wendy Caceres, MD, Hayley Gans, MD, Rebecca Blankenburg, MD, MPH

**Educational Objectives:**

By the end of this workshop, learners will be able to:

1. Define imposter syndrome to better recognize individuals and groups most impacted.
2. Discuss the prevalence and impact of imposter syndrome on the diversity of the medical workforce.
3. Develop tools to address imposter syndrome at the individual, peer, and institutional level.

**Detailed Agenda**

| **Amount of Time** | **Learning Objective** | **Learning Activity** |
| --- | --- | --- |
| 0-5 minutes |  | Introduction  Review Objectives |
| 5-15 minutes | Define imposter syndrome | Word cloud  Quiz  Video (optional) |
| 15-20 minutes |  | Brief didactic |
| 20- 30 minutes | Discuss the impact imposter syndrome has on: students, residents, fellows, faculty, staff | Small group case discussion |
| 30-45 minutes |  | Large group case discussion |
| 45- 55 minutes | Develop Tools 🡪Sponsorship. | Small group action plan development |
| 55- 70 minutes |  | Large group case discussion |
| 70- 75 minutes |  | Conclusion  Q & A  Evaluations |

**Supplies needed:**

1. Handouts (Appendix B)
2. PPT Slides (Appendix D)
3. Evaluation (Appendix E)

**Instructions for Young Imposter Syndrome Quiz**

“On page 1 of your handout, there is a Young Imposter Syndrome Quiz. Please answer these questions “yes” or “no” as honestly as possible. You won’t need to reveal your score unless you choose to.”

**Instructions for Small Group Cases**

“Now in small groups, you will have the opportunity to talk through one of six situations that illustrate how imposter syndrome can present itself or be amplified by others. Please talk through the case in your small groups, and answer the following questions:

1) Has something like this ever happened to you and how did it make you feel?

2) What are some factors that contribute to this happening?

In addition, please choose one member of your group to report your groups’ discussion in the large group.”

**Instructions for Large Group Discussion of Small Group Cases**

“We would now like to reconvene to our large group setting and discuss the small group cases. Let’s talk through them case by case. For each one, please share if something like this has happened to your group and how it made you feel. And share some factors that contributed to this happening.”

**Instructions for Small Group Discussion of Barriers and Strategies**

“Now in small groups, please discuss barriers and potential strategies to overcome these barriers at the individual, peer, and institutional levels. Please select someone in your group to share your group’s thoughts with the larger group.”

**Instructions for Large Group Discussion of Strategies**

“As a large group, we are now going to discuss potential strategies that you brainstormed to overcome these barriers at the individual, peer, and institutional levels.”

**Instructions for the Evaluation**

“We would love your feedback on how the workshop impacted your perspectives on imposter syndrome. Please fill out the anonymous evaluation.”
