# Supplementary material for: Do I Belong Here? Confronting Imposter Syndrome at an Individual, Peer, and Institutional Level in Health Professionals
Source: MedEdPORTAL. 2021 Jul 6;17:11166. doi: 10.15766/mep_2374-8265.11166 (PMC8257750; doi:10.15766/mep_2374-8265.11166)
Supplement: Supplementary file 1 — Facilitator Guide.docxWorkshop Handout.docxFacilitator Lesson Plan.docxPowerPoint Slides.pptxWorkshop Evaluation Form.docx [file mep_2374-8265.11166-s001.zip › B. Workshop Handout.docx]

**Young Imposter Syndrome Quiz (“Yes” or “No”)**

1. Do you secretly worry that others will find out you’re not as bright and capable as they think you are?
2. Do you sometimes shy away from challenges because of a nagging self-doubt?
3. Do you tend to chalk your accomplishments up to being a “fluke,” “no big deal” or the fact that people just “like” you?
4. Do you hate making a mistake, being less than fully prepared, or not doing things perfectly?
5. Do you tend to feel crushed even by constructive criticism, seeing it as evidence of your “ineptness?”
6. When you do succeed, do you think “Phew, I fooled them this time, but I may not be so lucky next time?”
7. Do you believe that other people (students, colleagues, competitors) are smarter and more capable than you?
8. Do you live in fear of being found out, discovered, or unmasked?

Responding “Yes” to five or more of these questions is considered a positive finding of Imposter Syndrome.

*Adapted from Villwock et. al., 2016*

Villwock JA, Sobin LB, Koester LA, Harris TM. Impostor syndrome and burnout among American medical students: a pilot study. *Int J Med Educ*. 2016;7:364-369.

**Case 1: Student**

You are a 19 year old college junior at a large state school sitting in a waiting room for an appointment with a campus career counselor to discuss applying to medical school. You have transferred from a community college and have been involved in multiple service projects for the largely underserved neighborhood where you are from. You meet a female student who has been working in a research lab and also considering applying to medical school. You start to wonder if you should even apply to medical school.

**Case 2: Resident**

You have been taking care of the same patient for 1 week, however in that time there have been 3 different attendings. The family asks you for updates on the plan as you are the team member that has been most consistent for them, however the team is not always keeping you in the loop as plans change. You begin to feel like you don’t have the authority or experience to update the family.

**Case 3: Fellow**

You are a senior fellow, currently applying for your first job. You arrive for an interview at a different institution to lead their new research study on a topic that you are extremely familiar with and have dedicated all of your career towards. When you arrive, the receptionist does not realize that you are here for an interview and thinks you are in the wrong place. Aware that you are from a minority group, unlike all the members of the practice, you begin to wonder if you deserve to be interviewed and that perhaps the invitation was a mistake.

**Case 4: Junior Faculty**

You are a new attending who recently started their first job. You recently graduated from your fellowship a few short months before in a field where you are an underrepresented minority. You were hired for a particular niche procedure with which you had developed exceptional skill. During a recent day, you were able to accomplish a technically difficult procedure, which was praised by the other physicians. However, you felt that you “just got lucky” and do not have the skills to perform your job well and confess this to another attending at a different institution.

**Case 5: Senior Faculty**

As a member of the faculty, you start to feel excluded in the department's activities. In meetings, you notice that others sit at the table, whereas you have never been invited to sit at the table. Others have been invited to serve on committees, but you never have been. Others' accomplishments are shared amongst the department, but your accomplishments are not celebrated in the same way. While relieved not to be the focus of attention, you are concerned about your ability to be promoted and wonder if perhaps you should seek a position at a different institution.

**Case 6: Staff**

You are an administrative assistant to the chair of the department, and you are tasked with a large project that is totally new to you in terms of previous experience. You are unsure of where to start the project and are apprehensive of asking questions. You arrive to a meeting not having completed the project and are asked why.

**The small groups should address the following two questions for each case:**

1. Has something like this ever happened to you and how did it make you feel?
2. What are some factors that contribute to this happening?

**Strategies to Overcome Imposter Syndrome**

Circle the action steps you will take in each category in the next week to overcome imposter syndrome.

**Institutional Level**

1. Institute regular feedback as a mechanism for all members of a team.
2. Start a formal mentorship program at each level
3. Sponsor diverse candidates for recruitment committees.
4. Start a “young attending” award to celebrate the accomplishments of younger faculty.

**Peer Level**

1. Educate your coworkers about imposter syndrome.
2. Recognize when others are confiding feelings of vulnerability and help them remember their worth.
3. Nominate your peer for well-deserved awards and honors.
4. Celebrate each others’ accomplishments.

**Individual Level**

1. Acknowledge feelings of imposter syndrome in yourself and remember how common these feelings are.
2. Confide these feelings in peers and mentors who can provide realistic and objective feedback.
3. Reframe to focus on your unique successes and skills.
4. Keep a list of your positive accomplishments that is easily accessible in times of self-doubt.
5. Ask to be on recruitment committees.

Notes:

______________________________________________________________________________

______________________________________________________________________________

______________________________________________________________________________

______________________________________________________________________________

**References:**

1. Clance PR, Imes SA. The imposter phenomenon in high achieving women: Dynamics and therapeutic intervention. *Psychotherapy: Theory, Research & Practice*. 1978;15(3):241-247.
2. Haney TS, Birkholz L, Rutledge C. A Workshop for Addressing the Impact of the Imposter Syndrome on Clinical Nurse Specialists. *Clin Nurse Spec*. 2018;32(4):189-194.
3. LaDonna KA, Ginsburg S, Watling C. “Rising to the Level of Your Incompetence”: What Physicians’ Self-Assessment of Their Performance Reveals About the Imposter Syndrome in Medicine. *Acad Med*. 2018;93(5):763-768.
4. Legassie J, Zibrowski EM, Goldszmidt MA. Measuring resident well-being: impostorism and burnout syndrome in residency. *J Gen Intern Med*. 2008;23(7):1090-1094.
5. Oriel K, Plane MB, Mundt M. Family medicine residents and the impostor phenomenon. *Fam Med*. 2004;36(4):248-252.
6. Villwock JA, Sobin LB, Koester LA, Harris TM. Impostor syndrome and burnout among American medical students: a pilot study. *Int J Med Educ*. 2016;7:364-369.
