# Supplementary material for: Do I Belong Here? Confronting Imposter Syndrome at an Individual, Peer, and Institutional Level in Health Professionals
Source: MedEdPORTAL. 2021 Jul 6;17:11166. doi: 10.15766/mep_2374-8265.11166 (PMC8257750; doi:10.15766/mep_2374-8265.11166)
Supplement: Supplementary file 1 — Facilitator Guide.docxWorkshop Handout.docxFacilitator Lesson Plan.docxPowerPoint Slides.pptxWorkshop Evaluation Form.docx [file mep_2374-8265.11166-s001.zip › C. Facilitator Lesson Plan.docx]

| **Amount of Time** | **Learning Objective** | **Learning Activity** | **Presenter** |
| --- | --- | --- | --- |
| 0-5 minutes |  | Introduction  Review Objectives |  |
| 5-15 minutes | Define imposter syndrome | Word cloud  Quiz  Video (optional) |  |
| 15-20 minutes |  | Brief didactic |  |
| 20- 30 minutes | Discuss the impact imposter syndrome has on: students, residents, fellows, faculty, staff | Small group case discussion |  |
| 30-45 minutes |  | Large group case discussion |  |
| 45- 55 minutes | Develop Tools 🡪Sponsorship. | Small group action plan development |  |
| 55- 70 minutes |  | Large group case discussion |  |
| 70- 75 minutes |  | Conclusion  Q & A  Evaluations |  |
