# Supplementary material for: Do I Belong Here? Confronting Imposter Syndrome at an Individual, Peer, and Institutional Level in Health Professionals
Source: MedEdPORTAL. 2021 Jul 6;17:11166. doi: 10.15766/mep_2374-8265.11166 (PMC8257750; doi:10.15766/mep_2374-8265.11166)
Supplement: Supplementary file 1 — Facilitator Guide.docxWorkshop Handout.docxFacilitator Lesson Plan.docxPowerPoint Slides.pptxWorkshop Evaluation Form.docx [file mep_2374-8265.11166-s001.zip › D. PowerPoint Slides.pptx]

## Slide 1
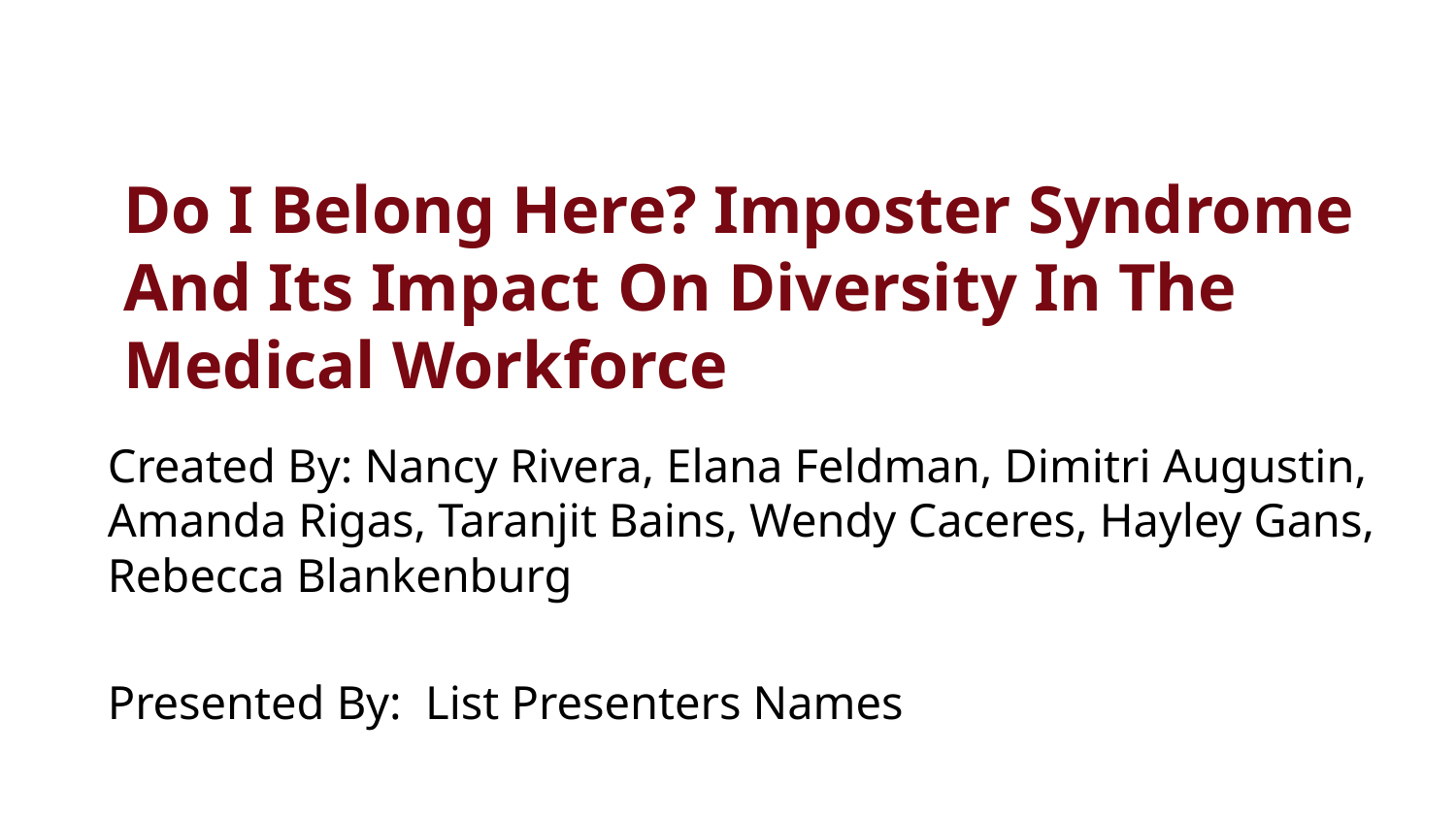

# Do I Belong Here? Imposter Syndrome And Its Impact On Diversity In The Medical Workforce
Created By: Nancy Rivera, Elana Feldman, Dimitri Augustin, Amanda Rigas, Taranjit Bains, Wendy Caceres, Hayley Gans, Rebecca Blankenburg
Presented By: List Presenters Names

## Slide 2
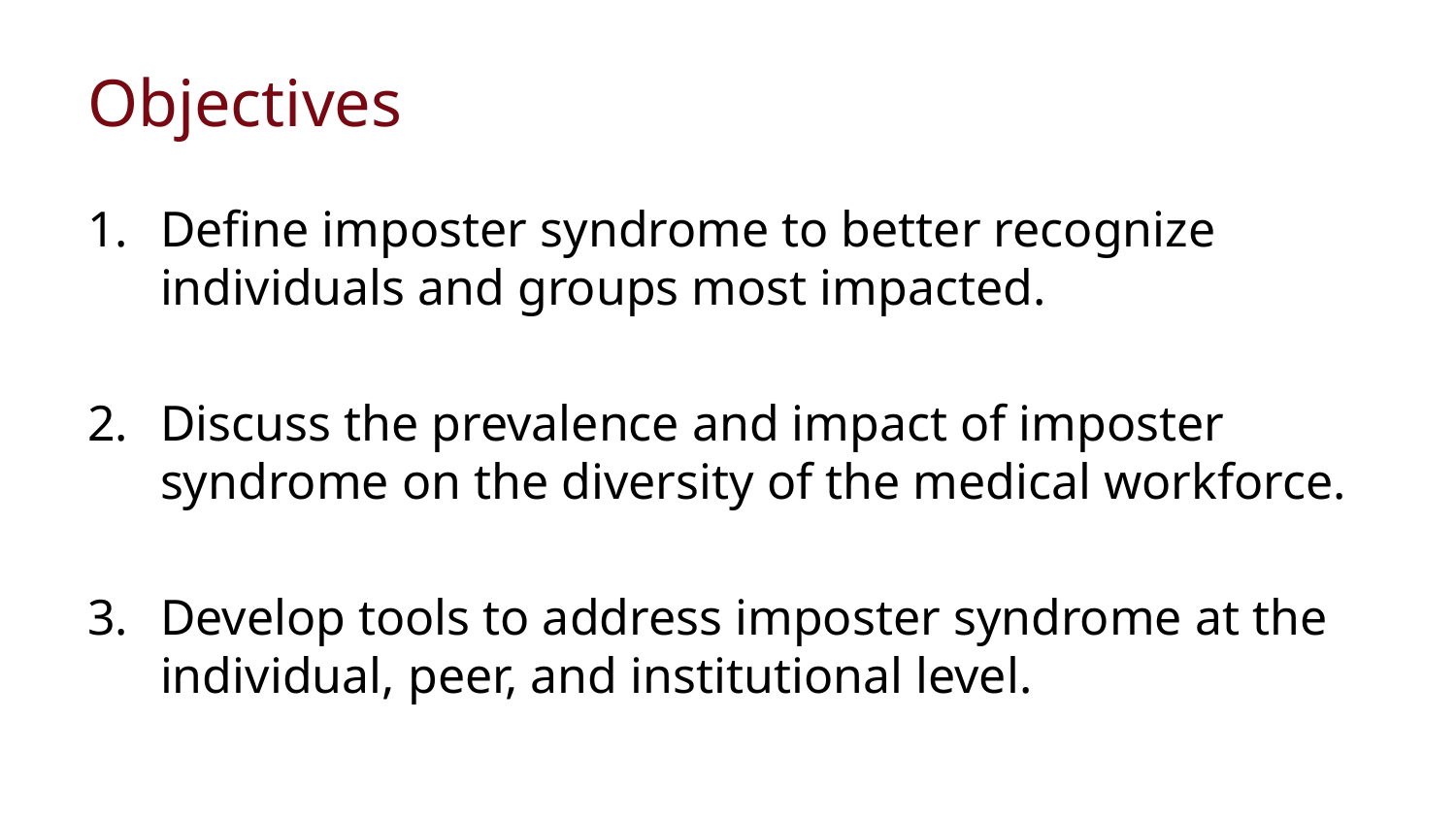

# Objectives
Define imposter syndrome to better recognize individuals and groups most impacted.
Discuss the prevalence and impact of imposter syndrome on the diversity of the medical workforce.
Develop tools to address imposter syndrome at the individual, peer, and institutional level.

## Slide 3
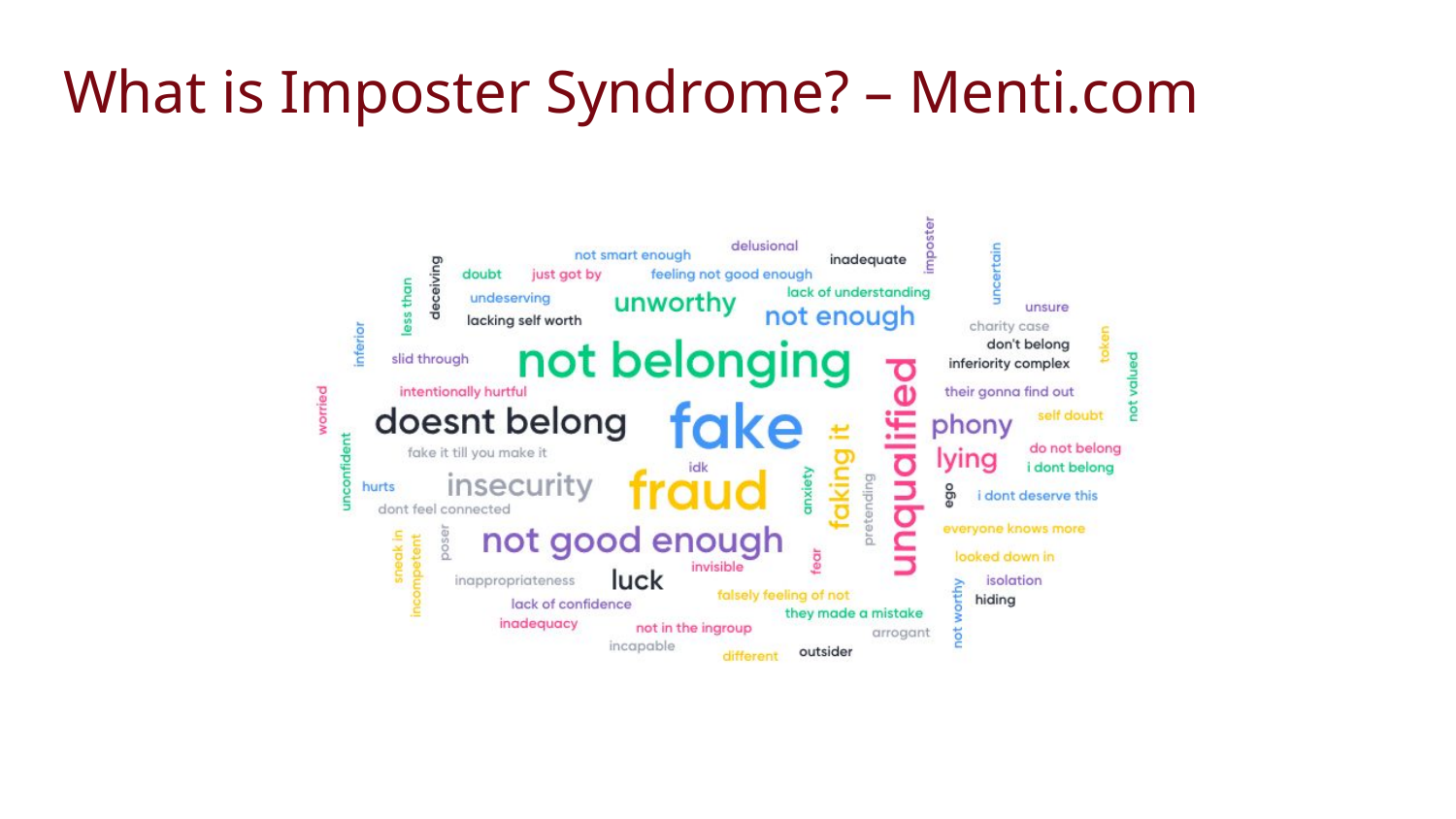

# What is Imposter Syndrome? – Menti.com

## Slide 4
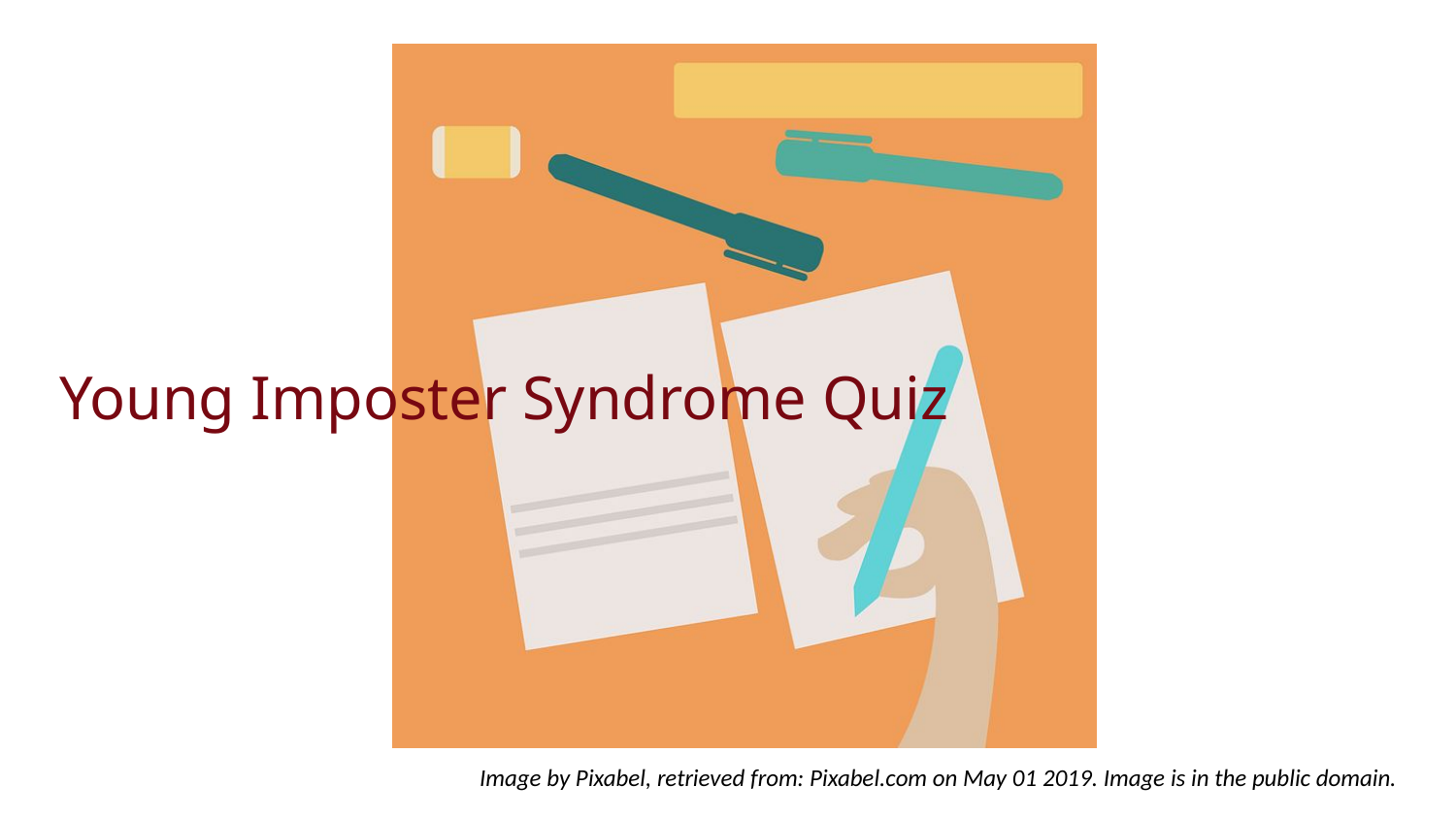

# Young Imposter Syndrome Quiz
Image by Pixabel, retrieved from: Pixabel.com on May 01 2019. Image is in the public domain.

## Slide 5
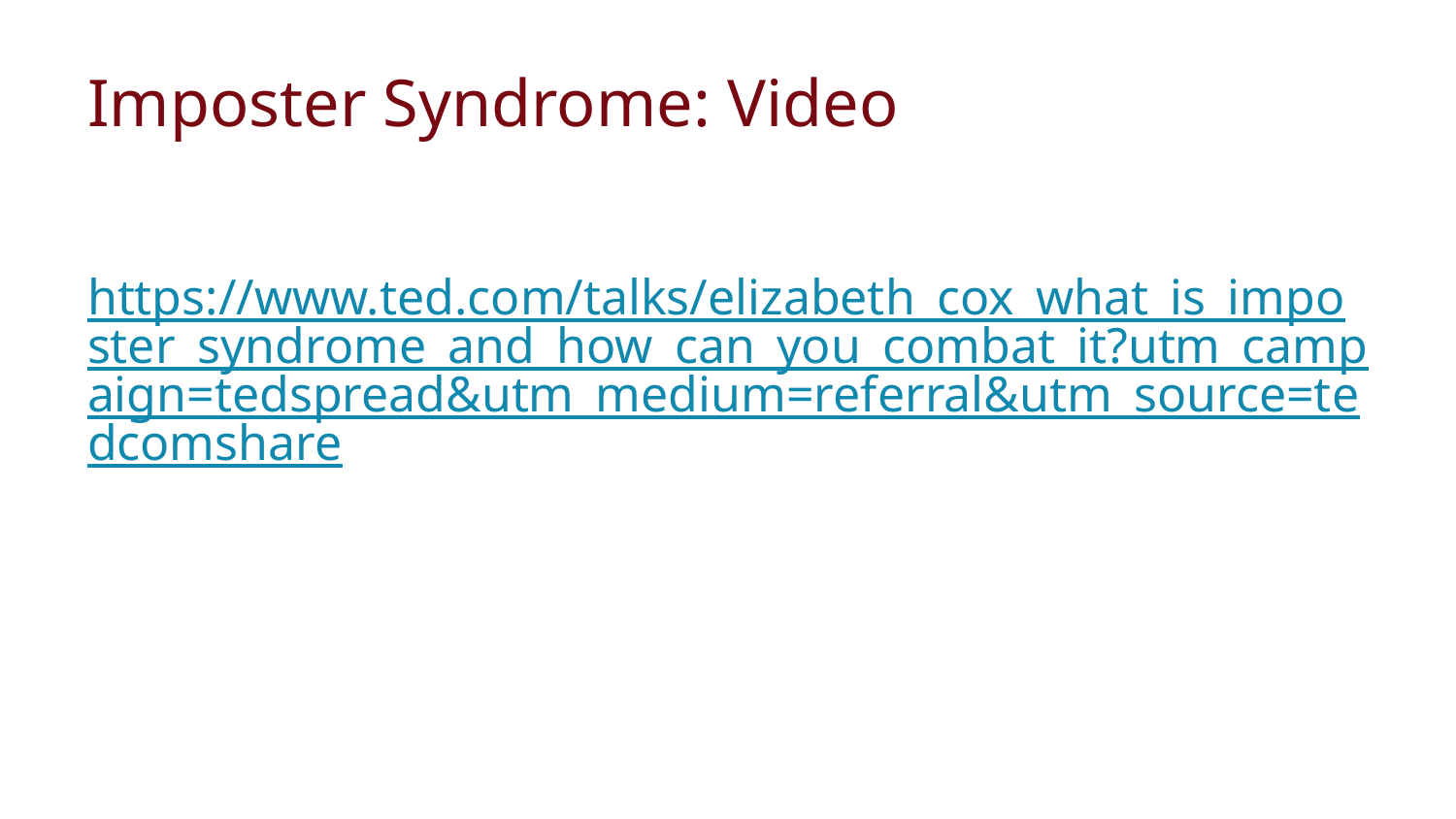

# Imposter Syndrome: Video
https://www.ted.com/talks/elizabeth_cox_what_is_imposter_syndrome_and_how_can_you_combat_it?utm_campaign=tedspread&utm_medium=referral&utm_source=tedcomshare

## Slide 6
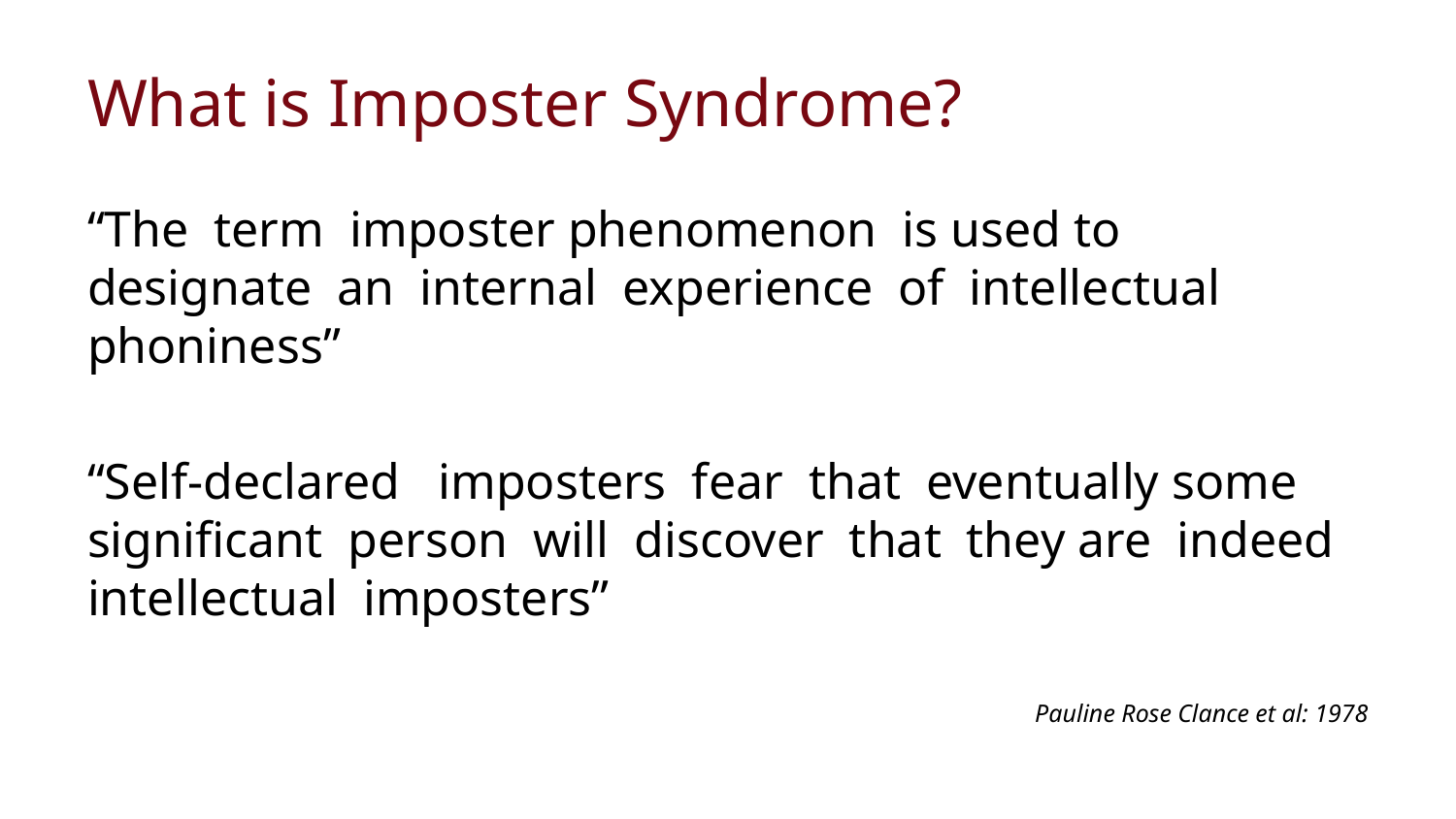

# What is Imposter Syndrome?
“The term imposter phenomenon is used to designate an internal experience of intellectual phoniness”
“Self-declared imposters fear that eventually some significant person will discover that they are indeed intellectual imposters”
Pauline Rose Clance et al: 1978

## Slide 7
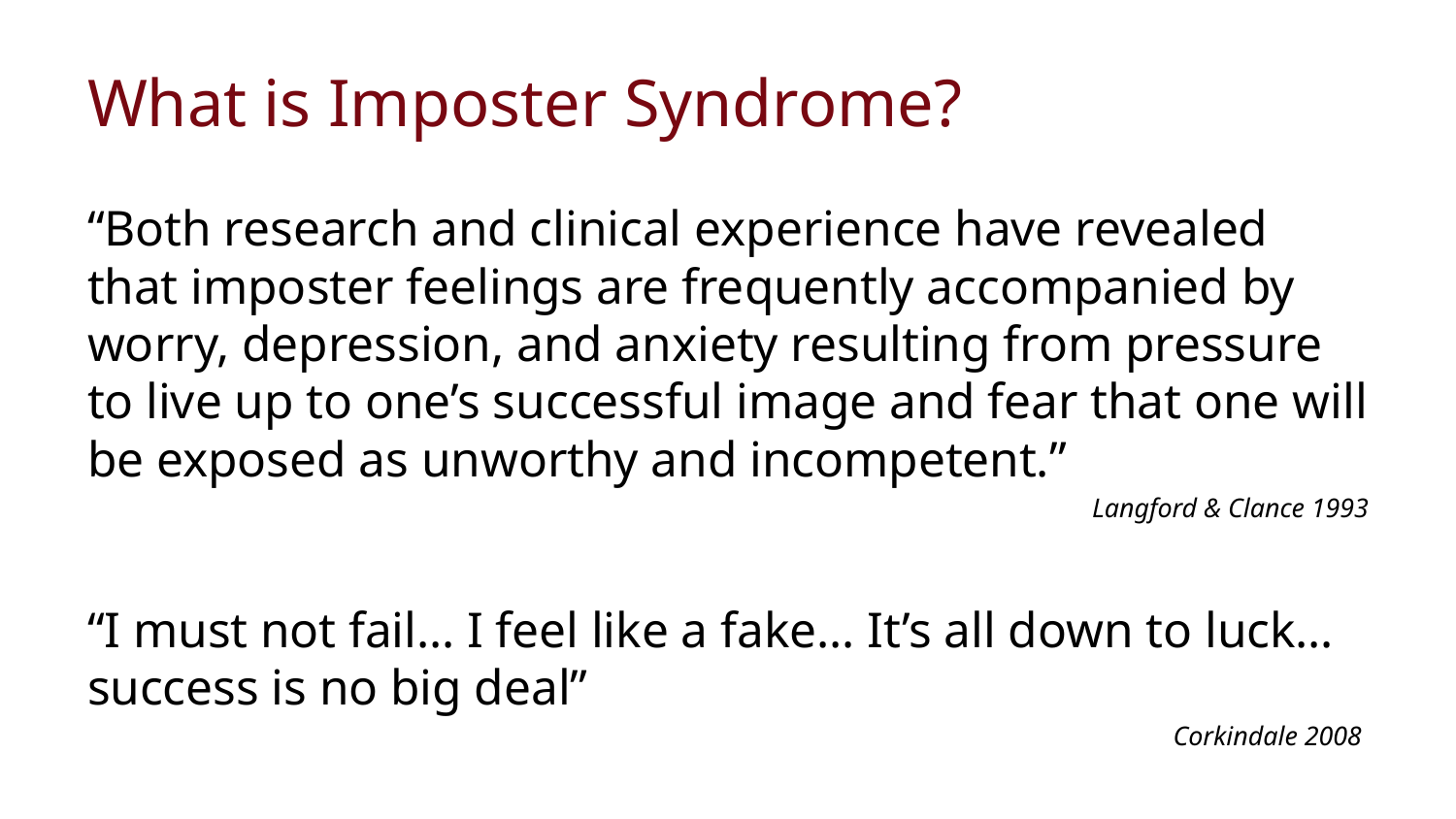

# What is Imposter Syndrome?
“Both research and clinical experience have revealed that imposter feelings are frequently accompanied by worry, depression, and anxiety resulting from pressure to live up to one’s successful image and fear that one will be exposed as unworthy and incompetent.”
Langford & Clance 1993
“I must not fail… I feel like a fake… It’s all down to luck… success is no big deal”
Corkindale 2008

## Slide 8
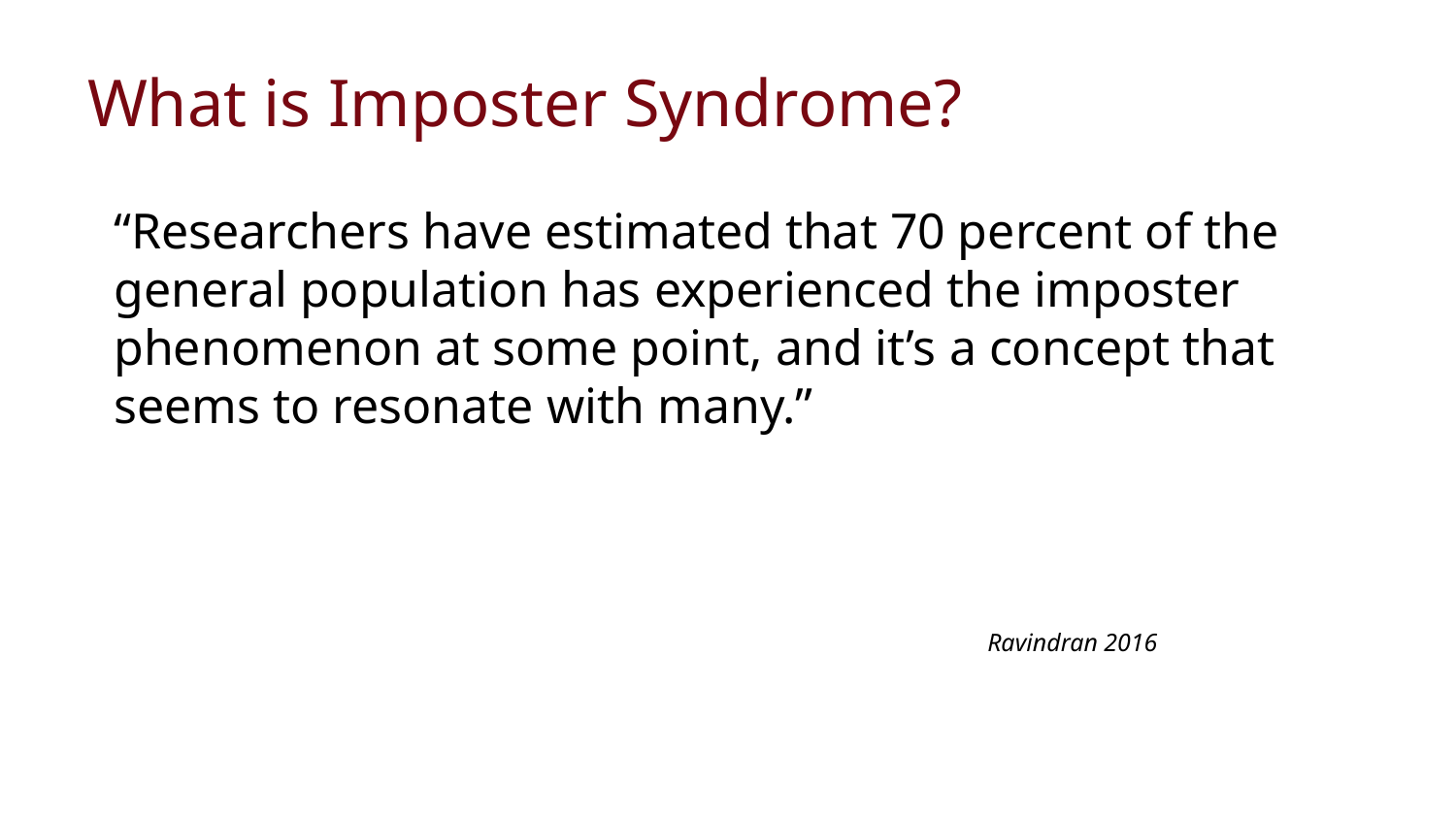

# What is Imposter Syndrome?
“Researchers have estimated that 70 percent of the general population has experienced the imposter phenomenon at some point, and it’s a concept that seems to resonate with many.”
														Ravindran 2016

## Slide 9
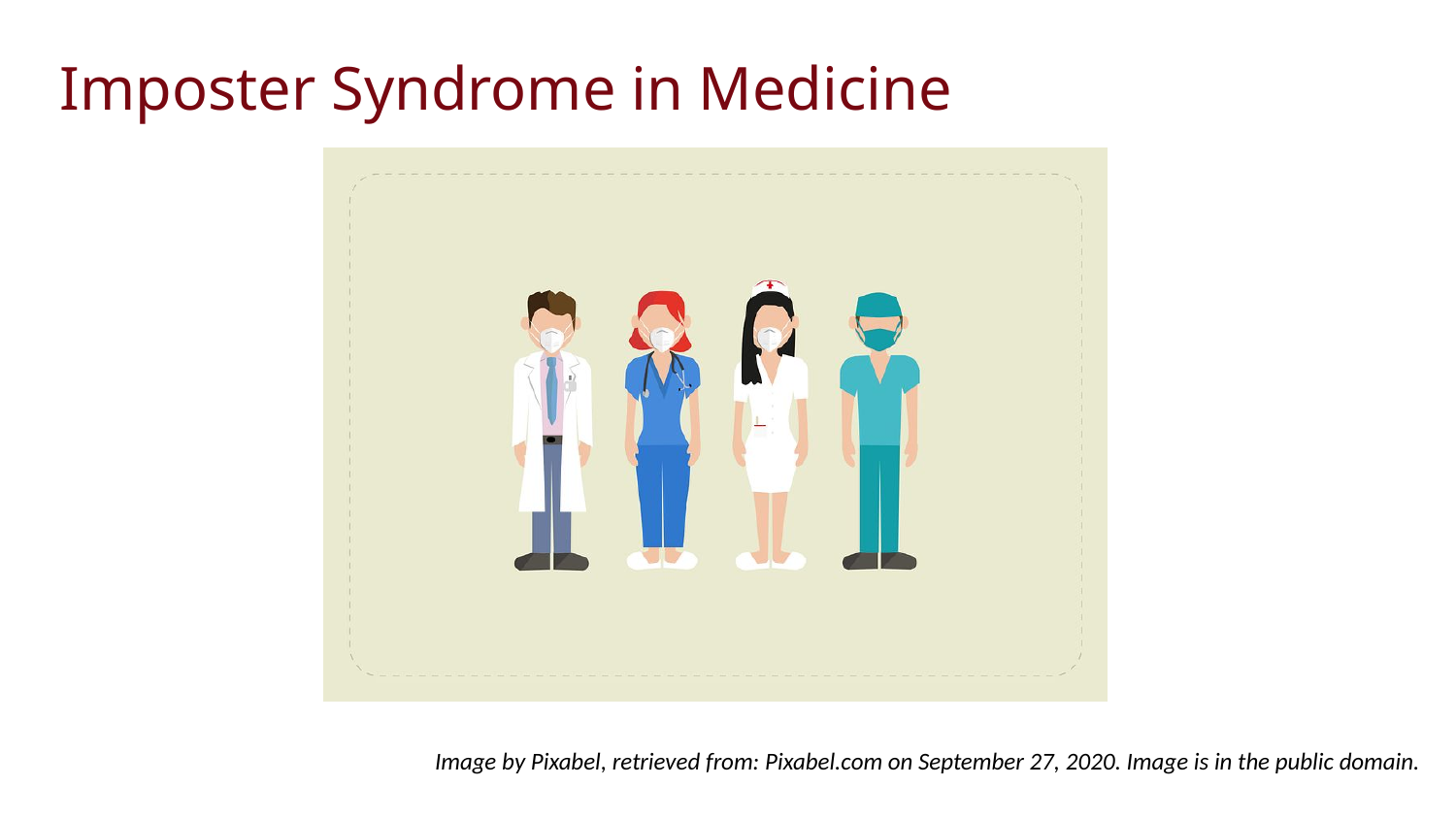

# Imposter Syndrome in Medicine
Image by Pixabel, retrieved from: Pixabel.com on September 27, 2020. Image is in the public domain.

## Slide 10
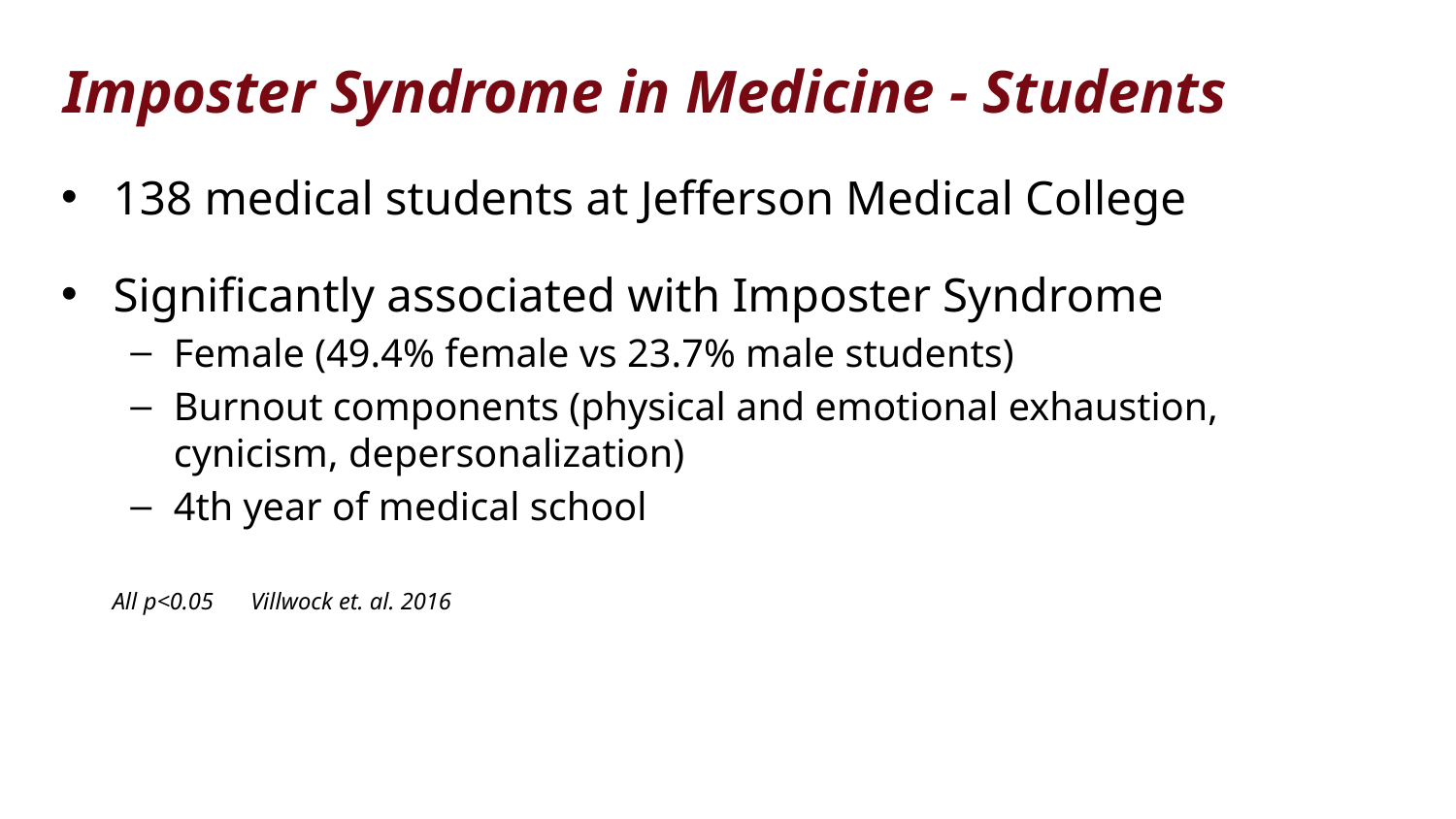

# Imposter Syndrome in Medicine - Students
138 medical students at Jefferson Medical College
Significantly associated with Imposter Syndrome
Female (49.4% female vs 23.7% male students)
Burnout components (physical and emotional exhaustion, cynicism, depersonalization)
4th year of medical school
												All p<0.05		Villwock et. al. 2016

## Slide 11
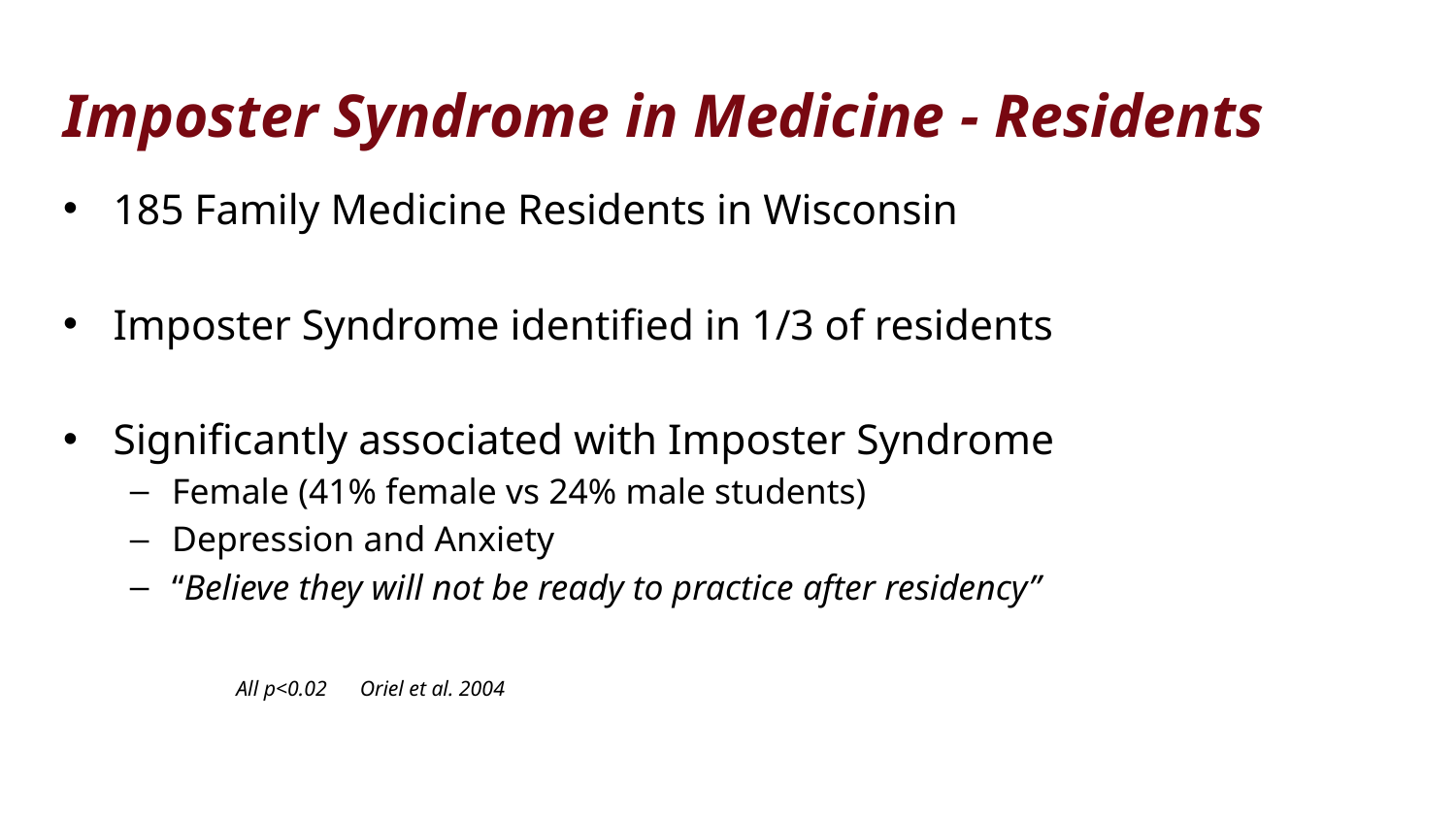

# Imposter Syndrome in Medicine - Residents
185 Family Medicine Residents in Wisconsin
Imposter Syndrome identified in 1/3 of residents
Significantly associated with Imposter Syndrome
Female (41% female vs 24% male students)
Depression and Anxiety
“Believe they will not be ready to practice after residency”
										 	All p<0.02		Oriel et al. 2004

## Slide 12
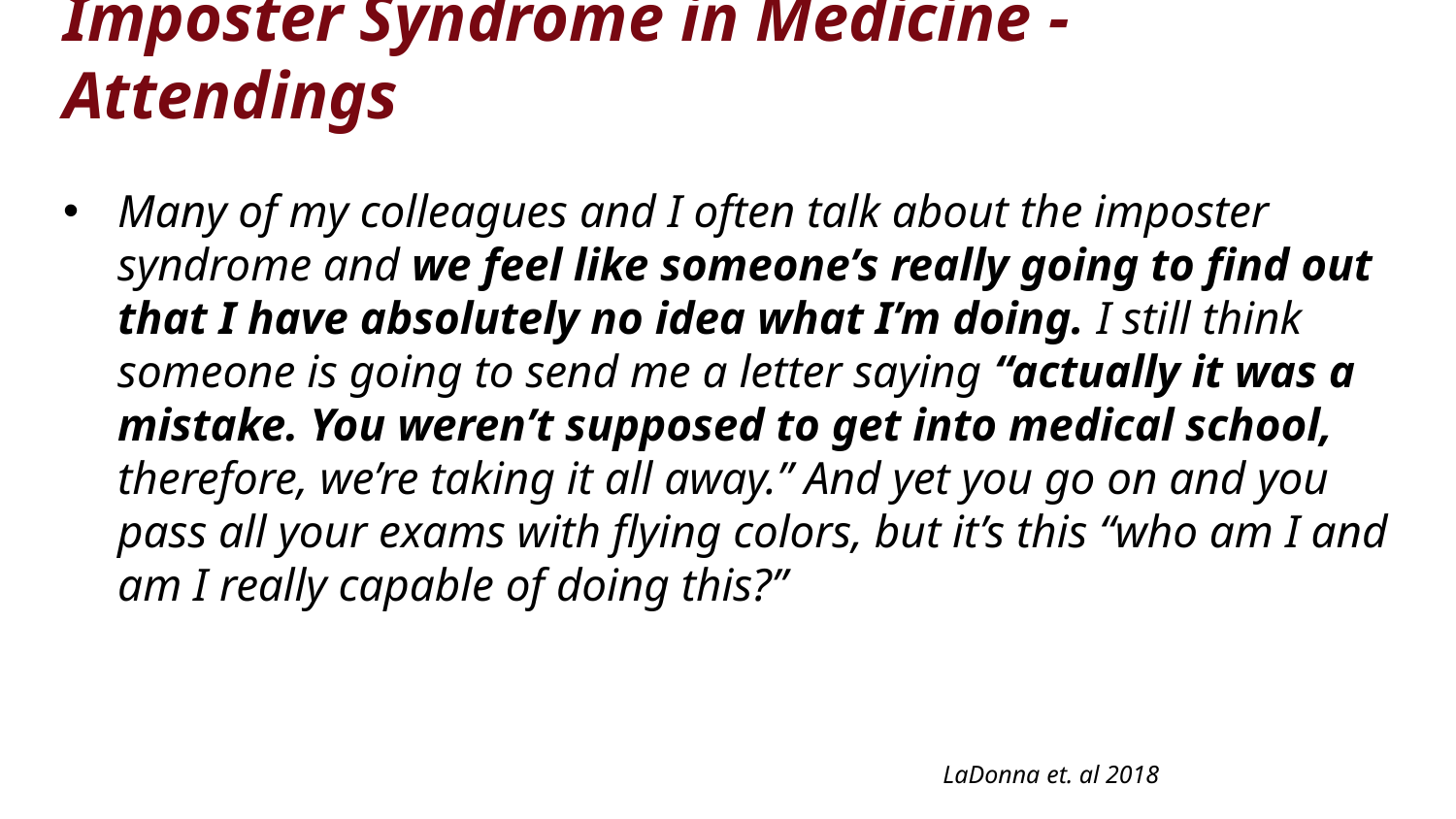

# Imposter Syndrome in Medicine - Attendings
Many of my colleagues and I often talk about the imposter syndrome and we feel like someone’s really going to find out that I have absolutely no idea what I’m doing. I still think someone is going to send me a letter saying “actually it was a mistake. You weren’t supposed to get into medical school, therefore, we’re taking it all away.” And yet you go on and you pass all your exams with flying colors, but it’s this “who am I and am I really capable of doing this?”
															 LaDonna et. al 2018

## Slide 13
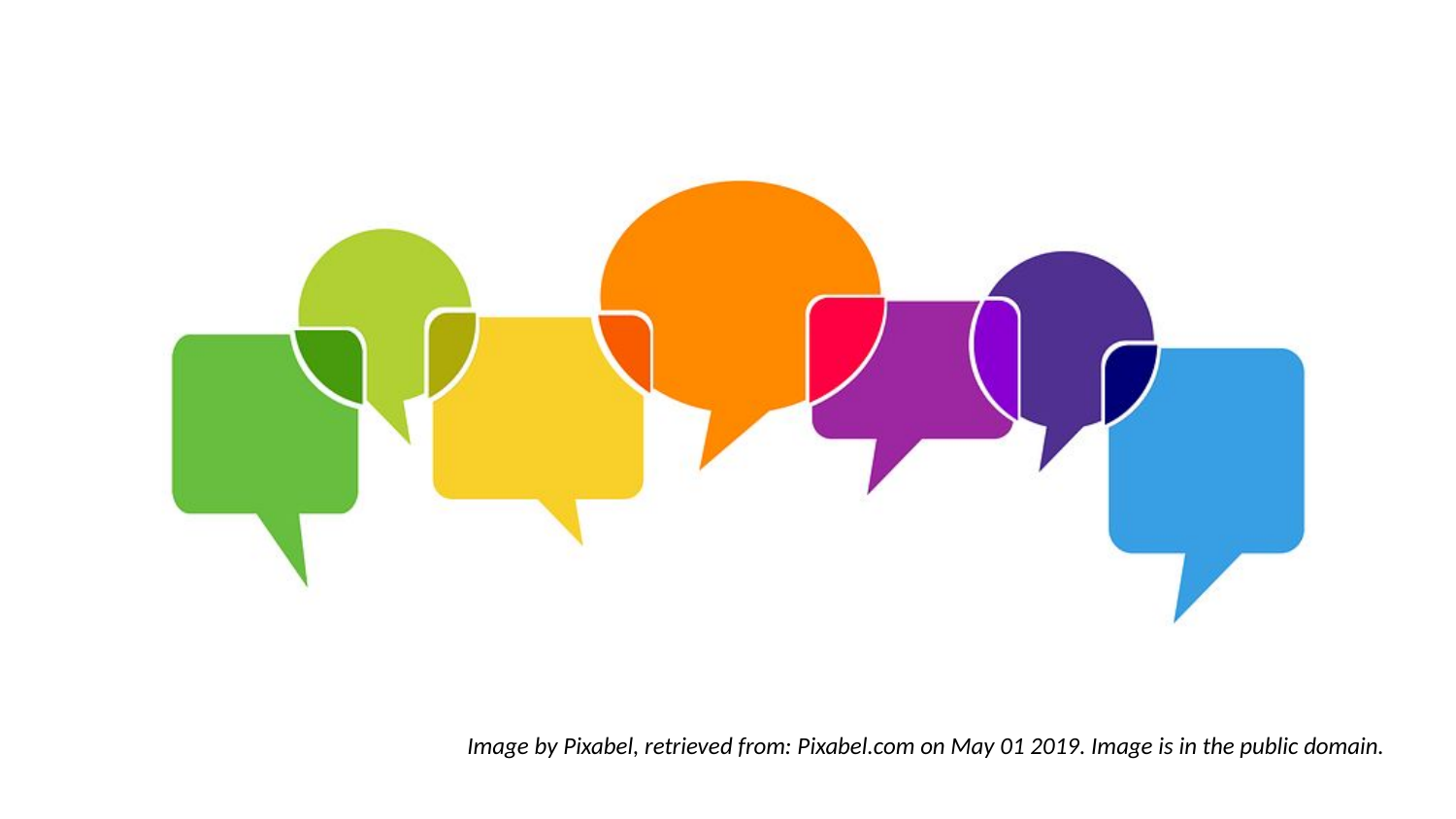

Image by Pixabel, retrieved from: Pixabel.com on May 01 2019. Image is in the public domain.

## Slide 14
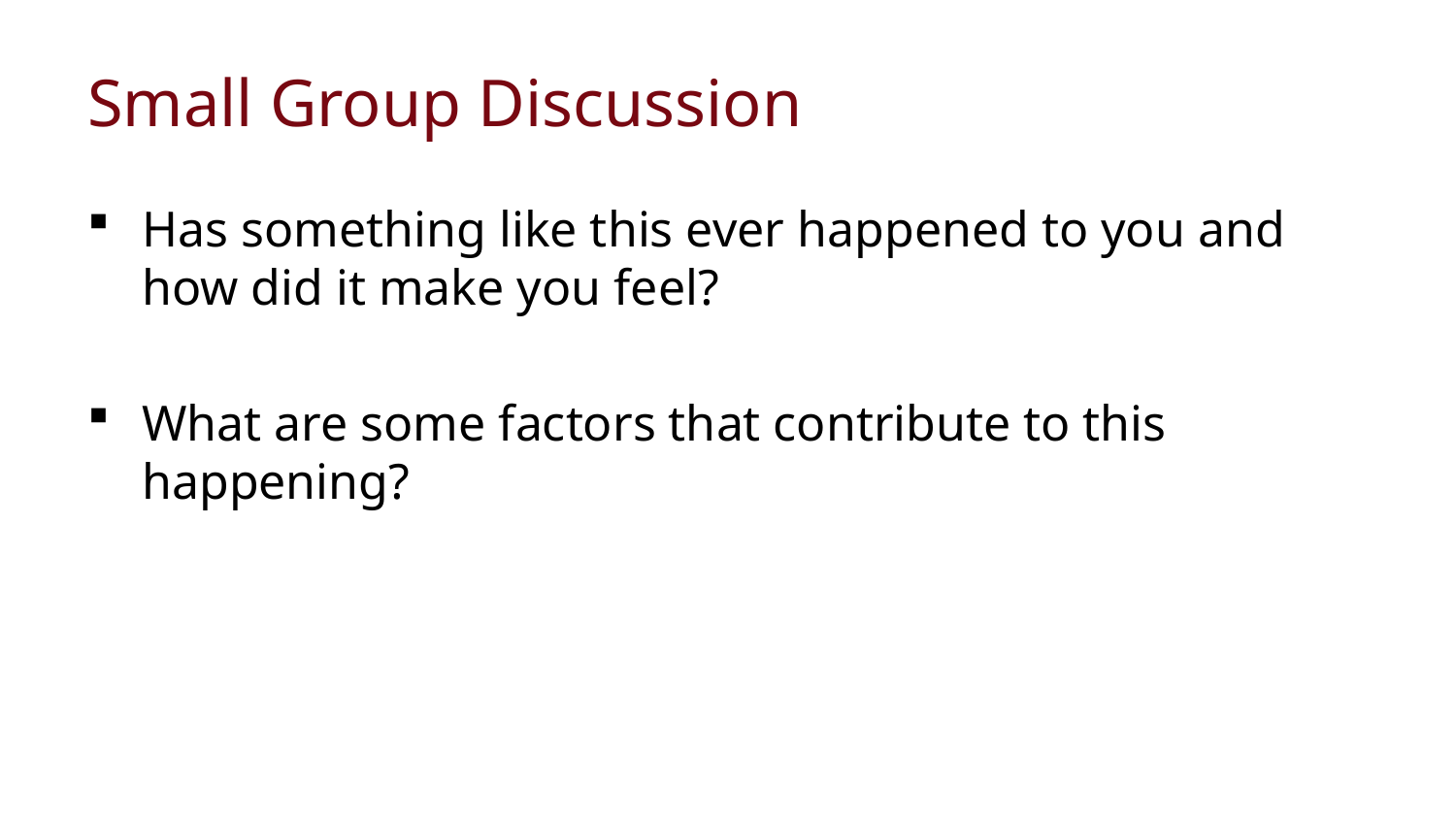

# Small Group Discussion
Has something like this ever happened to you and how did it make you feel?
What are some factors that contribute to this happening?

## Slide 15
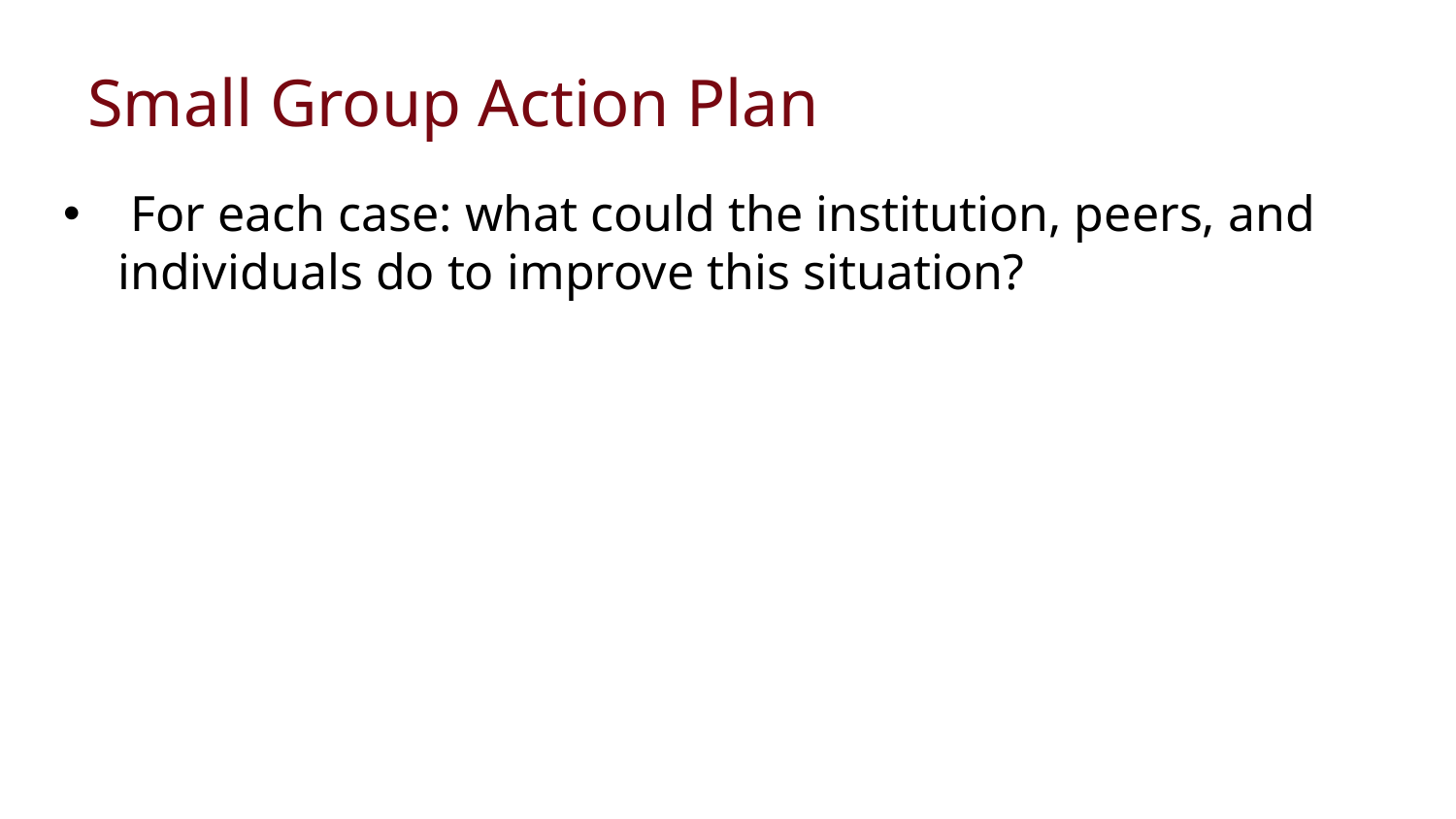

# Small Group Action Plan
 For each case: what could the institution, peers, and individuals do to improve this situation?

## Slide 16
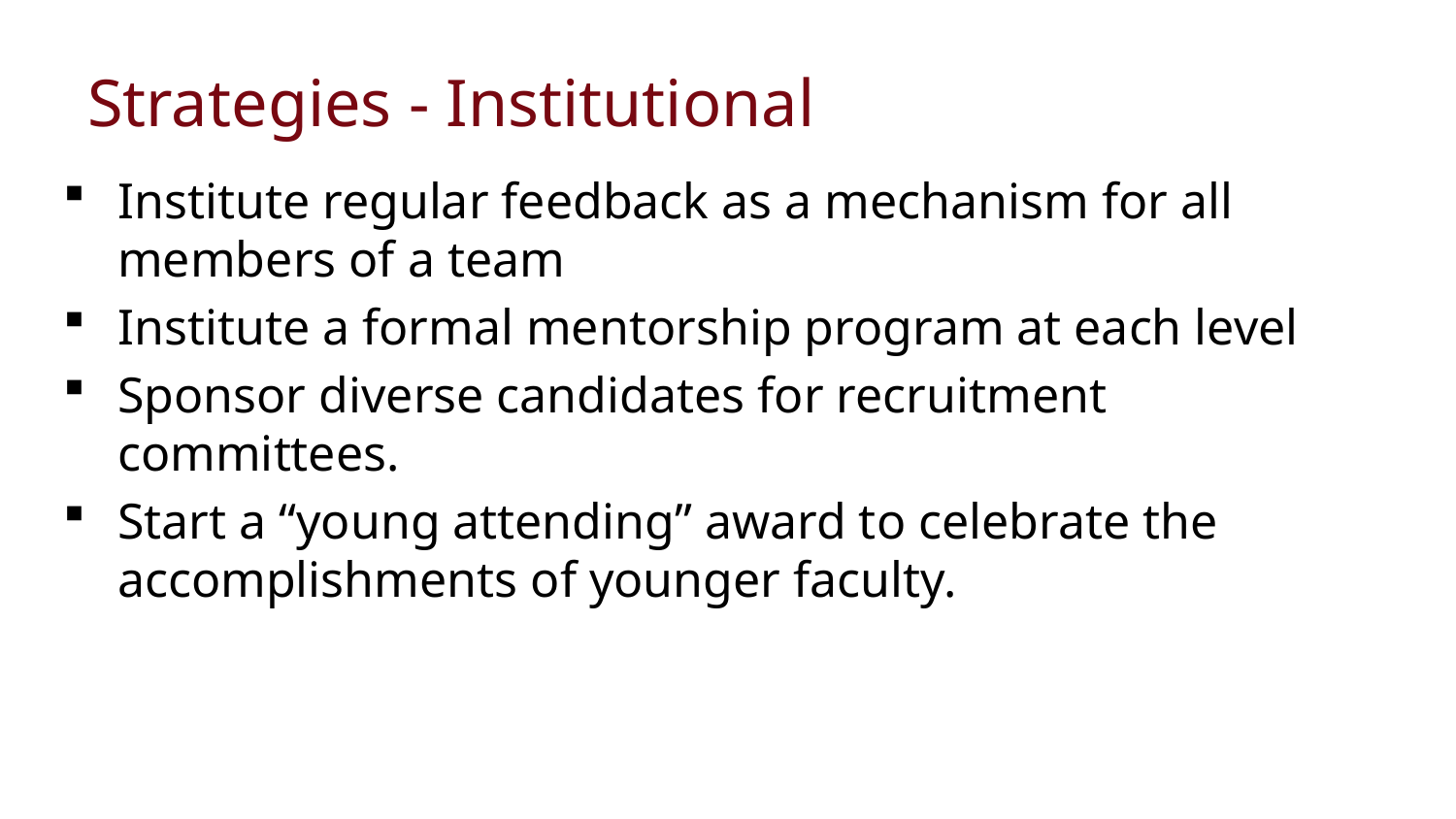

# Strategies - Institutional
Institute regular feedback as a mechanism for all members of a team
Institute a formal mentorship program at each level
Sponsor diverse candidates for recruitment committees.
Start a “young attending” award to celebrate the accomplishments of younger faculty.

## Slide 17
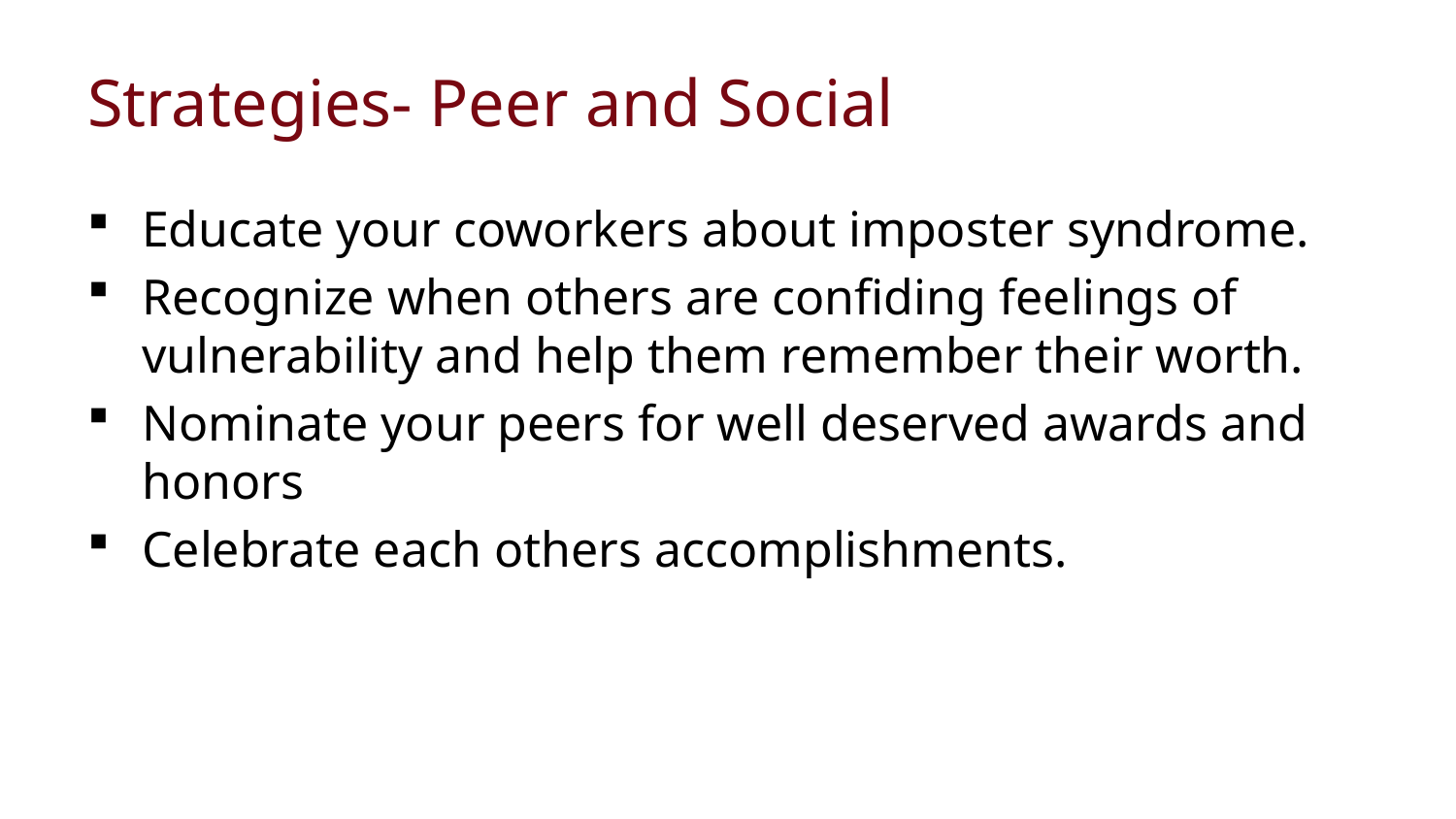

# Strategies- Peer and Social
Educate your coworkers about imposter syndrome.
Recognize when others are confiding feelings of vulnerability and help them remember their worth.
Nominate your peers for well deserved awards and honors
Celebrate each others accomplishments.

## Slide 18
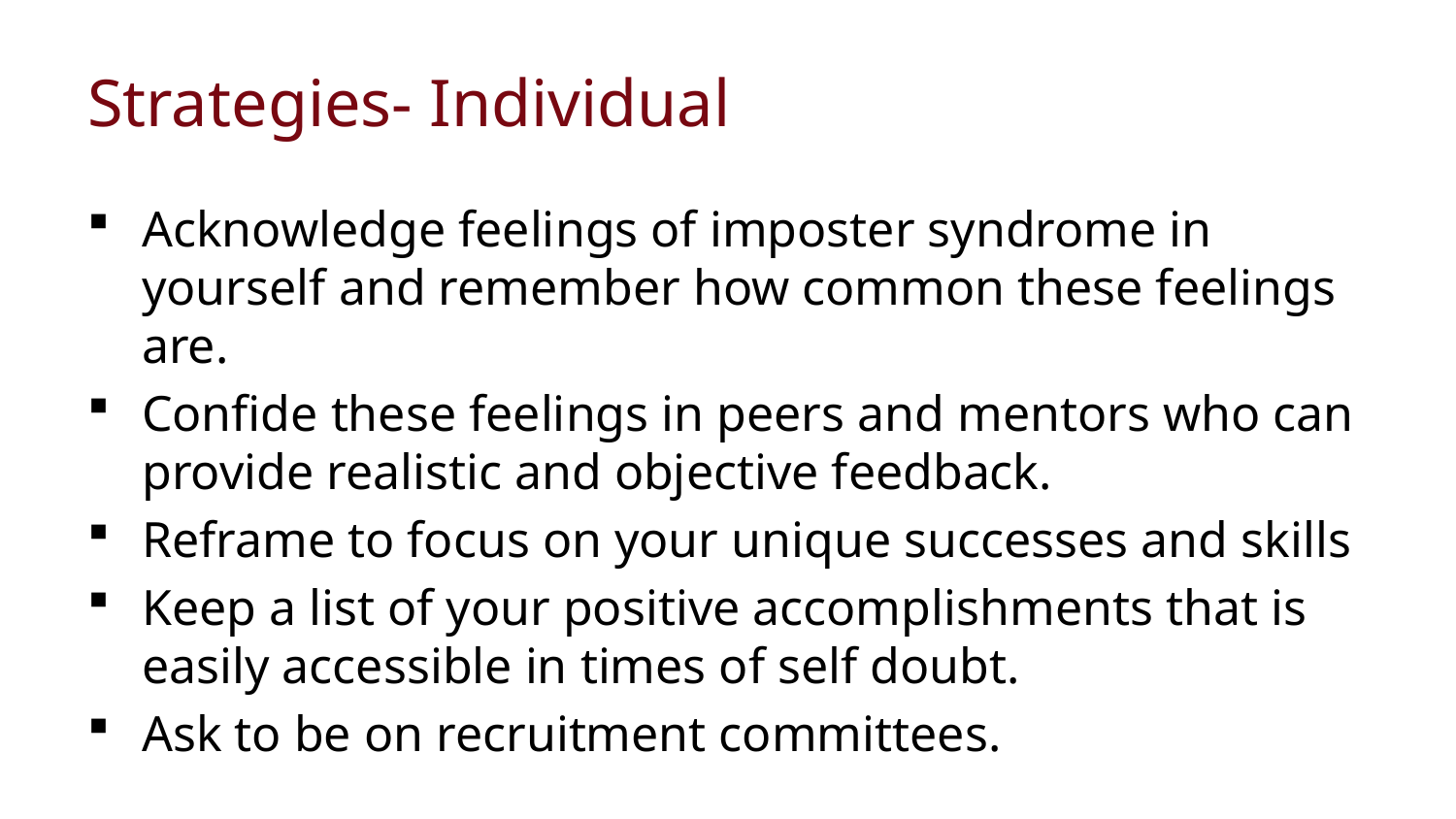

# Strategies- Individual
Acknowledge feelings of imposter syndrome in yourself and remember how common these feelings are.
Confide these feelings in peers and mentors who can provide realistic and objective feedback.
Reframe to focus on your unique successes and skills
Keep a list of your positive accomplishments that is easily accessible in times of self doubt.
Ask to be on recruitment committees.

## Slide 19
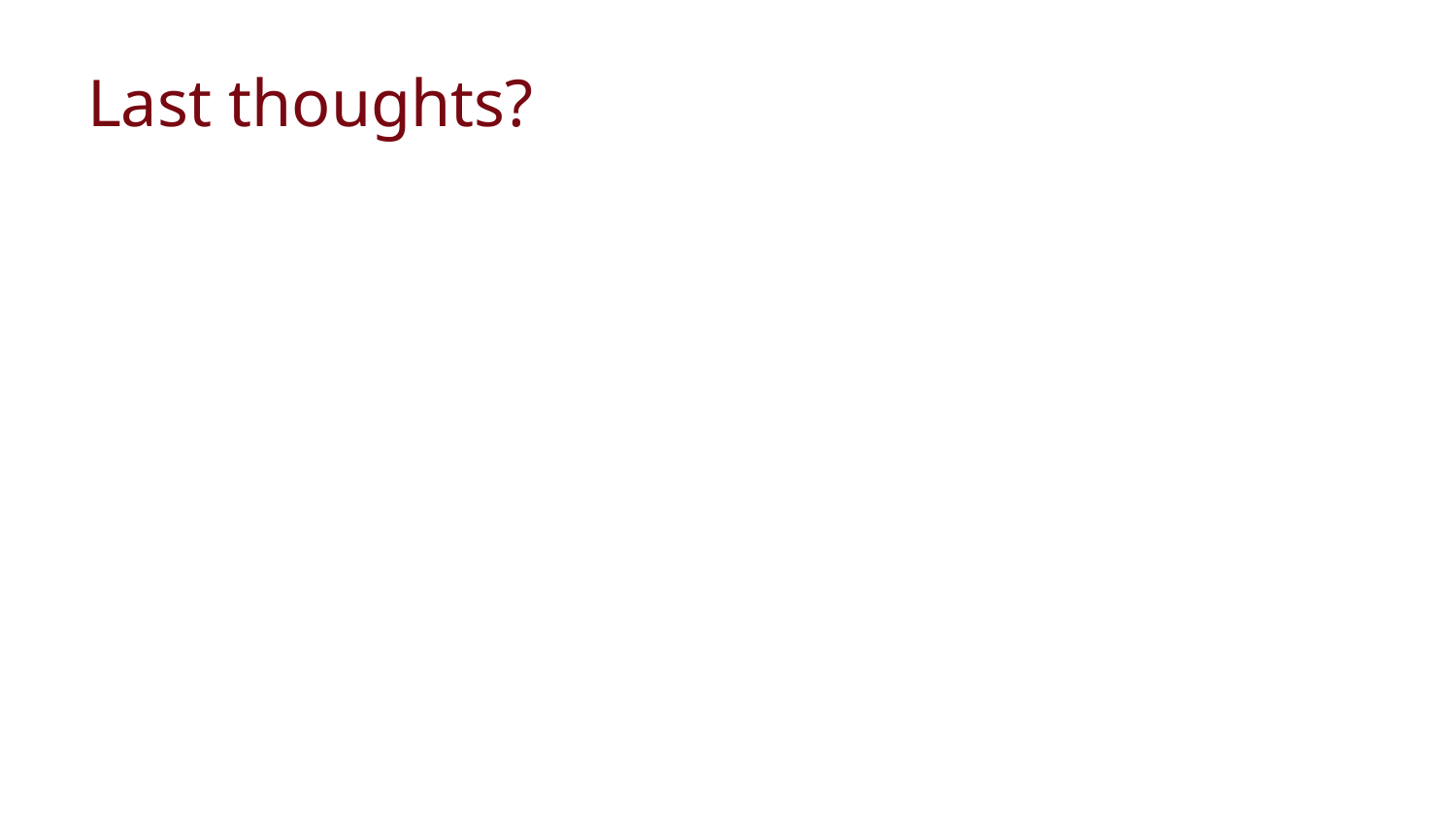

# Last thoughts?

## Slide 20
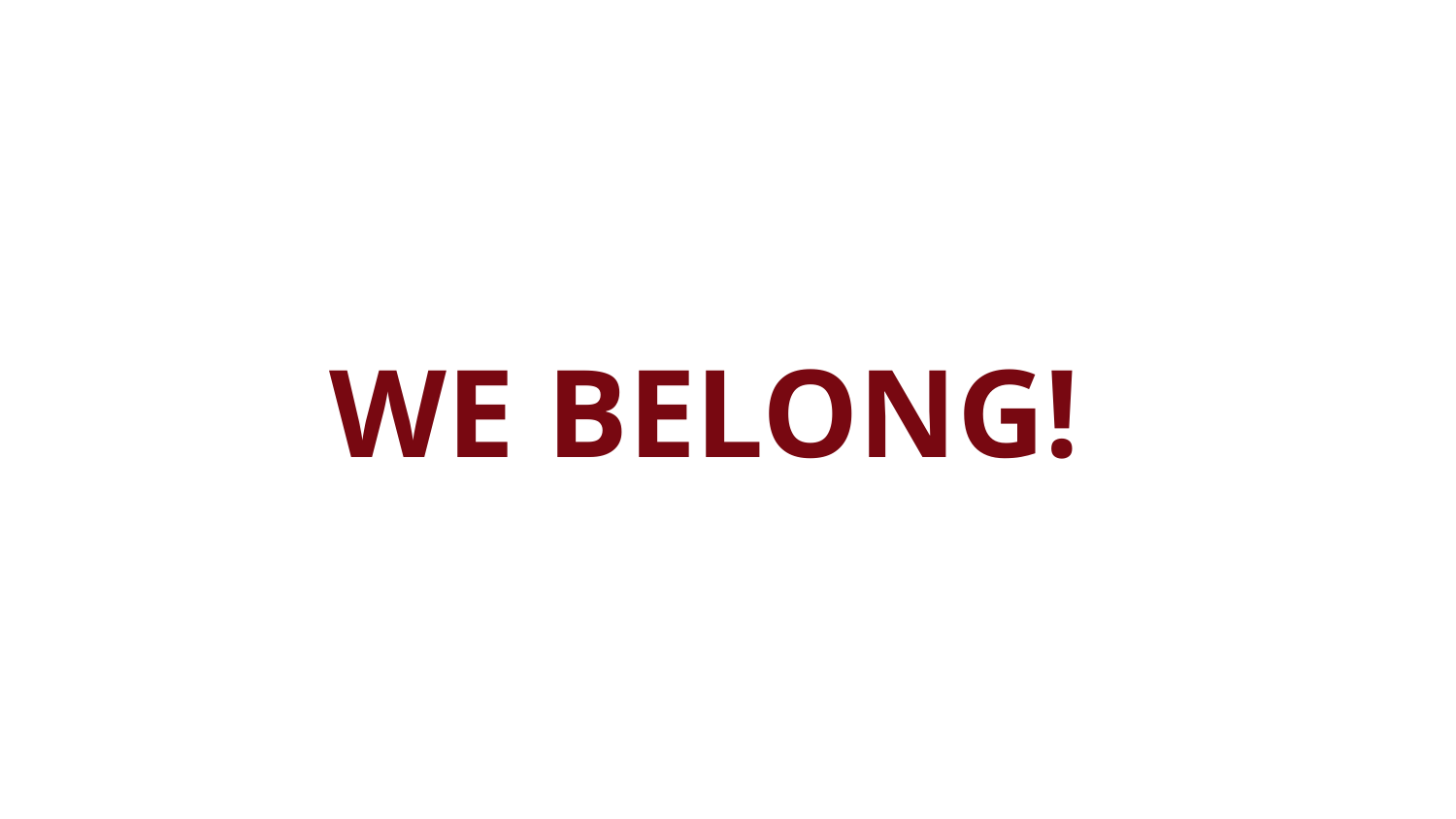

# WE BELONG!

## Slide 21
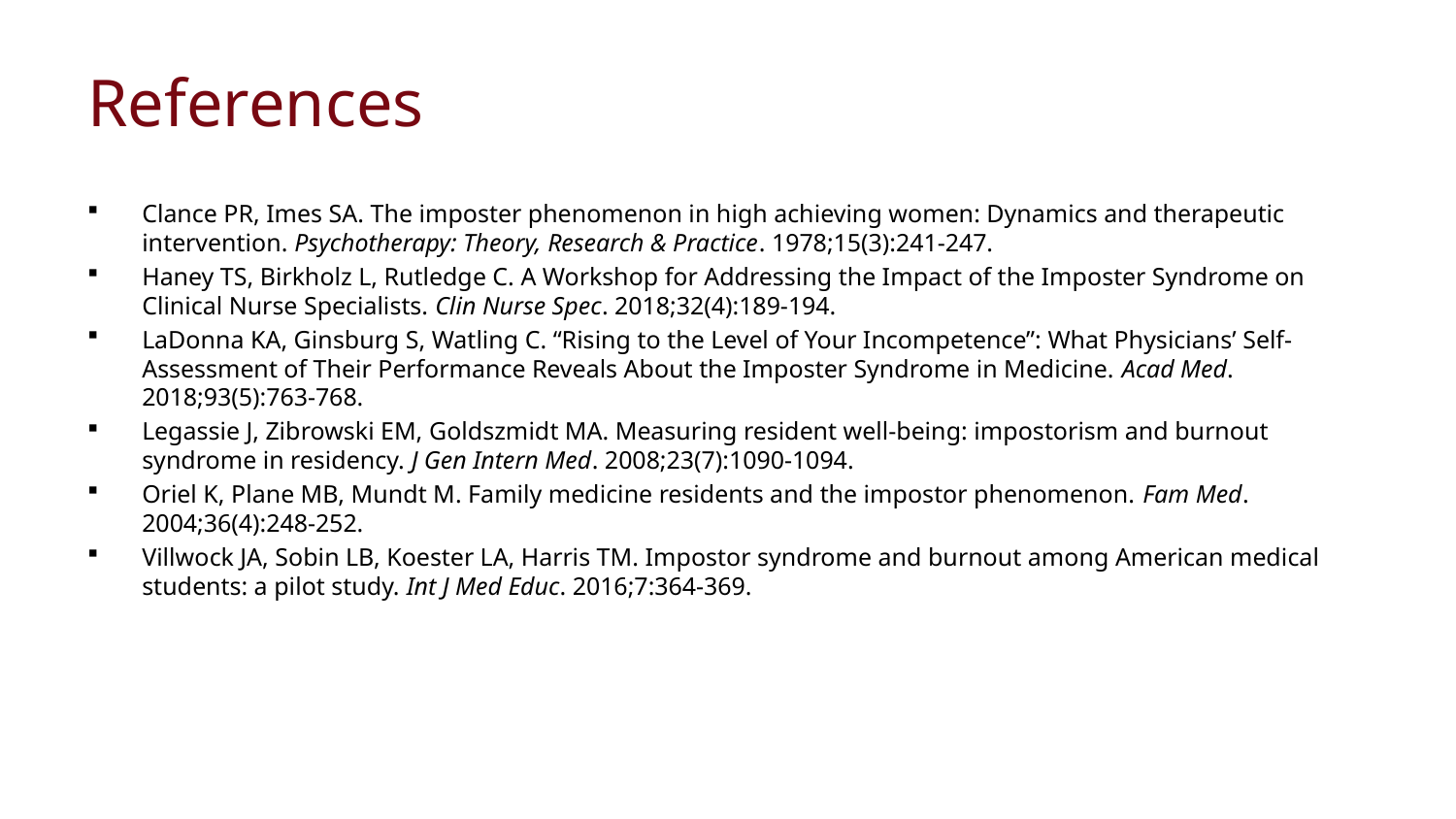

# References
Clance PR, Imes SA. The imposter phenomenon in high achieving women: Dynamics and therapeutic intervention. Psychotherapy: Theory, Research & Practice. 1978;15(3):241-247.
Haney TS, Birkholz L, Rutledge C. A Workshop for Addressing the Impact of the Imposter Syndrome on Clinical Nurse Specialists. Clin Nurse Spec. 2018;32(4):189-194.
LaDonna KA, Ginsburg S, Watling C. “Rising to the Level of Your Incompetence”: What Physicians’ Self-Assessment of Their Performance Reveals About the Imposter Syndrome in Medicine. Acad Med. 2018;93(5):763-768.
Legassie J, Zibrowski EM, Goldszmidt MA. Measuring resident well-being: impostorism and burnout syndrome in residency. J Gen Intern Med. 2008;23(7):1090-1094.
Oriel K, Plane MB, Mundt M. Family medicine residents and the impostor phenomenon. Fam Med. 2004;36(4):248-252.
Villwock JA, Sobin LB, Koester LA, Harris TM. Impostor syndrome and burnout among American medical students: a pilot study. Int J Med Educ. 2016;7:364-369.
