# Supplementary material for: Do I Belong Here? Confronting Imposter Syndrome at an Individual, Peer, and Institutional Level in Health Professionals
Source: MedEdPORTAL. 2021 Jul 6;17:11166. doi: 10.15766/mep_2374-8265.11166 (PMC8257750; doi:10.15766/mep_2374-8265.11166)
Supplement: Supplementary file 1 — Facilitator Guide.docxWorkshop Handout.docxFacilitator Lesson Plan.docxPowerPoint Slides.pptxWorkshop Evaluation Form.docx [file mep_2374-8265.11166-s001.zip › E. Workshop Evaluation Form.docx]

**Do I Belong Here? Imposter Syndrome and Its Impact on Diversity in the Medical Workforce**

**Add Date Here**

**Add Conference Title**

**EVALUATION FORM**

**Workshop Objectives:**

1. Define imposter syndrome to better recognize individuals and groups most impacted.
2. Discuss the prevalence and impact of imposter syndrome on the diversity of the medical workforce.
3. Develop tools to address imposter syndrome at the individual, peer, and institutional level.

**Strongly disagree Neutral Strongly agree**

Workshop met objectives 1 2 3 4 5

Workshop was a valuable use of my time 1 2 3 4 5

Handouts include useful resources 1 2 3 4 5

I will apply information learned today to 1 2 3 4 5

address imposter syndrome

What two things will you do as a result of this workshop?

1.

2.

What do you see as potential barriers to applying what you have learned?

What did you like best about the workshop?

What can we improve about the workshop?
