# Supplementary material for: Do I Belong Here? Confronting Imposter Syndrome at an Individual, Peer, and Institutional Level in Health Professionals
Source: MedEdPORTAL. 2021 Jul 6;17:11166. doi: 10.15766/mep_2374-8265.11166 (PMC8257750; doi:10.15766/mep_2374-8265.11166)
Supplement: Supplementary file 1 — Facilitator Guide.docxWorkshop Handout.docxFacilitator Lesson Plan.docxPowerPoint Slides.pptxWorkshop Evaluation Form.docx [file mep_2374-8265.11166-s001.zip › Educational_Summary_Report_11166.pdf]

MedEdPORTAL® The AAMC Journal of Teaching and Learning Resources

Original Publication Open Access

Do I Belong Here? Confronting Imposter Syndrome at an Individual, Peer, and Institutional Level in Health Professionals

Nancy Rivera, MD, MS*, Elana A. Feldman, MD, Dimitri A. Augustin, MD, Wendy Caceres, MD, Hayley A. Gans, MD, Rebecca Blankenburg, MD, MPH

*Corresponding Author: nrivera3@stanford.edu

Abstract

Introduction: Imposter syndrome (IS) is a feeling of being an intellectual fraud and is common among health professionals, particularly those underrepresented in medicine. IS is accompanied by burnout, self-doubt, and beliefs of decreased success. This workshop aims to discuss the impact of IS and develop strategies to confront IS at the individual, peer, and institutional levels. **Methods:** During the 75-minute interactive workshop, participants listened to didactics and engaged in individual reflection, small-group case discussion, and large-group instruction. Workshop participants and facilitators included medical students, residents, fellows, faculty, staff, and program leadership. Anonymous postworkshop evaluations exploring participants' satisfaction and intentions to change their behavior were collected. Descriptive statistics were used to analyze the quantitative data, and content analysis was used to analyze participants' intentions to change their behavior. **Results:** The workshop was presented at three local academic conferences and accepted at one national conference. Data were collected from 92 participants. Ninety-two percent of participants felt the workshop met its objectives, and 90% felt the workshop was a valuable use of their time. Furthermore, 90% of participants stated they would apply information learned at the workshop in the future. The participants indicated an intent to change behavior on individual, peer, and institutional levels, while recognizing that barriers exist at all those levels. **Discussion:** This workshop proved to be an effective means to discuss strategies on how to address IS at the individual, peer, and institutional levels. The materials can be adapted for relevance to various audiences.

Keywords
Imposter Syndrome, Underrepresented in Medicine (UIM), Workshop, Case-Based Learning, Interprofessional Education, Diversity, Inclusion, Health Equity

Educational Objectives

By the end of this workshop, learners will be able to:

1. Define imposter syndrome to better recognize individuals and groups most impacted.
2. Discuss the prevalence and impact of imposter syndrome on individuals historically underrepresented in medicine.
3. Develop tools to address imposter syndrome at the individual, peer, and institutional levels.

Citation:
Rivera N, Feldman EA, Augustin DA, Caceres W, Gans HA, Blankenburg R. Do I belong here? Confronting imposter syndrome at an individual, peer, and institutional level in health professionals. *MedEdPORTAL*. 2021;17:11166.
https://doi.org/10.15766/mep_2374-8265.11166

Introduction

Imposter syndrome (IS) is the internalized feeling of self-doubt and not belonging in a particular group that can lead to the fear of being discovered as a fraud. It was first described in a population of high-achieving women by Clance and Imes in 1978.[1] Since then, this phenomenon has been increasingly recognized, with an estimated 70% of the general population having experienced IS at some point.[2]

Importantly, IS is common within the medical profession. It is known to impact all levels of medical training, beginning with medical students[3] and continuing during stages of transition[4] and even in senior faculty.[5] Additionally, studies have explored how self-identifying with an underrepresented group in medicine, including racial, ethnic, gender, LGBTQ, religious, and other minorities, contributes to IS.[3,6-9]

At the individual level, IS has been associated with burnout, emotional and physical exhaustion, depression, and anxiety.[3]

Copyright © 2021 Rivera et al. This is an open-access publication distributed under the terms of the Creative Commons Attribution license.

Furthermore, IS fosters self-doubt and impacts ability to receive feedback, resiliency, well-being, and success. At the systemic level, those who experience IS may be less likely to apply for promotions for fear of failure, leading to decreased diversity in leadership positions, such as deans or department chairs within academic medicine.[10] To effectively promote diversity, equity, and inclusion within academic medicine, IS must be recognized, acknowledged, and confronted on both individual and systemic levels to strengthen the pipeline of diverse candidates.

Effective tools and strategies to identify and confront IS are very limited; however, some interventions have been implemented within the health care professions. A facilitator-guided 30- to 45-minute intervention on 21 internal medicine residents was published in *MedEdPORTAL*.[11] This intervention included self-reflection, think-pair-share, and large-group discussion; 81% of the participating residents felt the intervention promoted resident wellness. A 3-hour educational session with new medical interns involved small groups, role-playing, and facilitator feedback.[12] It was well received by the trainees and helped them realize that they could ask others for help and were not alone in their feeling of fear. A 1-day interprofessional educational workshop where participants took the Clance Impostor Phenomenon Scale (CIPS), listened to didactics, and engaged in discussion helped clinical nurse specialist students feel a sense of liberation and empowerment.[13] An online module consisting of a 14-minute educational video with supplemental reminder cards of proposed coping mechanisms was presented to a cohort of dental students.[14] Based on pre- and postmodule CIPS responses, there was a decrease in IS scores at the end of the semester. These interventions show the strength of modules and workshops in improving awareness of IS and providing strategies that help reduce the impact of IS in health care professionals.

While studies have provided evidence that interventions to address IS are effective in reducing feelings of IS, clear tools and strategies that focus on IS and diversity are lacking. This module provides an interprofessional framework for medical trainees, faculty, staff, and program leadership to understand, acknowledge, and confront IS while promoting diversity, equity, and inclusion through interactive didactics, group sessions, and strategies directed at IS that may change behavior.

Methods

Workshop

The workshop was developed using Kern's six-steps of curriculum development[15]:

1. Problem identification and general needs assessment: The curriculum was designed to address IS, which is common among health professionals, has been described at higher rates in groups that are underrepresented in medicine, and can lead to individuals feeling as if they do not belong in academic medicine. An extensive literature search was done, including reviewing peer-reviewed manuscripts and curricula (including *MedEdPORTAL*).
2. Targeted needs assessment: A lecture presented for the Stanford Leadership Education in Advancing Diversity Program's 2018 cohort revealed shared feelings of IS. Studies describing tools and strategies focused on overcoming IS and promoting diversity were lacking.
3. Goals and objectives:
   - Define IS to better recognize individuals and groups most impacted.
   - Discuss the prevalence and impact of IS on individuals historically underrepresented in medicine.
   - Develop tools to address IS at the individual, peer, and institutional levels.
4. Educational strategies: In order to engage the audience, a mixture of direct and interactive learning was used. Interactive methods included using word clouds, small-group discussion, and large-group discussion.
5. Implementation: The formal workshop comprised didactics, individual reflection, small-group case discussion, and large-group instruction that correlated with Bloom's taxonomy.[16]
6. Evaluating the effectiveness of the curriculum: Immediately after the workshop, participants completed an evaluation assessing its effectiveness in meeting objectives.

The workshop was aimed at participants of all levels, including students, residents, fellows, faculty, staff, and program leadership. It was designed as a 75-minute session; however, the timing could be amended to meet the specific needs of the audience. The workshop could be incorporated during orientations, retreats, and diversity, equity, and inclusion conferences; across all health care professions; and at various institutions, including academic and community hospitals and clinics.

Workshop facilitators could be residents, fellows, faculty, staff, and program leadership. In a 60-minute training session, facilitators were taught about the IS phenomenon, definition, evidence-based research, and potential strategies to assist with feelings of IS. Facilitators' roles were assigned beforehand and included sharing personal narratives, leading didactics, facilitating small-group discussions, guiding large-group instruction, and assisting with logistics during the workshop (Appendix A).

Copyright © 2021 Rivera et al. This is an open-access publication distributed under the terms of the Creative Commons Attribution license.

Small-group sizes were ideally six to eight participants. As they entered the room, participants were divided into small groups—either randomized groups or separated by professional role (i.e., student-only group, resident-only group, staff-only group, faculty-only group). The layout of the room, the number of participants, and the different learner levels were factors that assisted in determining which small-group format was ideal to meet the workshop needs. Randomized groups composed of individuals with different levels of training and expertise provided diverse perspectives when discussing the small-group cases, whereas groups divided by professional designation fostered an environment of comradery and vulnerability given that other individuals might be experiencing the same issues discussed at the very same time.

As the workshop started, the facilitators shared their personal experiences of IS, which created an environment where vulnerability was respected and invited. The facilitators then discussed the learning objectives of the workshop.

Participants were asked to reflect on their own thoughts and experiences concerning IS by participating in an interactive word cloud and completing a validated IS quiz (Appendix B). The word cloud was created via Menti.com and asked participants, “What is imposter syndrome? If you don’t know, you may answer ‘IDK’.” If unable to create a word cloud through Menti.com, an alternative would be to have participants raise their hands to answer the question while the facilitator writes their responses on a board. The validated IS quiz was the Young Impostor Scale (YIS), an abbreviated eight-question quiz using yes-or-no statements.[3] Responding yes to five or more of these questions was considered a positive finding of IS, which provided real-time individualized and private feedback to the participants. The participants were asked to discuss their perspectives and reflections on both activities. Facilitators then led a short didactic on IS, which included an optional showing of a 2-minute TED Talk video excerpt on IS[17] and shared evidence-based research on the effects of IS in medical students, residents, and attendings.

Following this introduction to IS and participants’ reflection on its impact on their own lives, the workshop continued with interactive discussion of cases in facilitated small groups, followed by large-group discussions. Small-group cases were developed based on personal experiences of the authors and their colleagues and were created to reflect experiences with which the participants could identify. Depending on the number of small groups and how they were divided, each group was assigned one to two cases (Appendix B). Once participants had read their assigned cases and the first two discussion questions to themselves, each small group discussed the cases together. Then, small groups shared their reflections with the entire group, with one facilitator leading the discussion and another facilitator assisting with the audience responses.

For the last discussion question, small groups reconvened and developed strategies to overcome IS on the individual, peer, and institution levels. Facilitators led this large-group instruction, combining the strategies the small groups had suggested with evidence-based and expert consensus-driven strategies developed for the workshop (Appendix B). Afterward, postworkshop evaluations were collected from all participants.

The workshop time line (with materials) was as follows:

- Preworkshop preparation:
  - One hour of facilitator training; materials: facilitator guide (Appendix A).
- Workshop lesson plan and PowerPoint slides: (Appendices C and D, respectively):
  - 0-5 minutes: introductions and review of learning objectives; materials: PowerPoint slides (Appendix D).
  - 5-15 minutes: interactive word cloud, validated assessment, and TED Talk video excerpt.[17] Materials for online word cloud: cell phone or tablet with internet access; materials for the YIS quiz: workshop handout (Appendix B); materials for TED Talk video (optional): internet access to stream excerpt of video.
  - 15-20 minutes: brief didactic; materials: PowerPoint slides (Appendix D).
  - 20-30 minutes: small-group case-based discussion; materials: workshop handout (Appendix B).
  - 30-45 minutes: large-group debrief of cases; materials: workshop handout (Appendix B).
  - 45-55 minutes: small-group action plan development; materials: workshop handout (Appendix B).
  - 55-70 minutes: large-group debrief for action plan; materials: workshop handout (Appendix B).
  - 70-75 minutes: conclusion with Q&A, evaluations; materials: evaluation forms (Appendix E).

Workshop Evaluation

As the workshop concluded, participants were asked to complete the anonymous postworkshop evaluation form (Appendix E). This evaluation used four items rated on a 5-point Likert scale (1 = *strongly disagree,* 5 = *strongly agree*) plus four open-ended questions to evaluate the effectiveness of the workshop, including participant satisfaction and intended behavior change.

Copyright © 2021 Rivera et al. This is an open-access publication distributed under the terms of the Creative Commons Attribution license.

Analysis
Descriptive statistics were used to analyze the quantitative data (percentage of participants who strongly agreed or agreed). Content analysis was used to analyze the participants' intentions to change their behavior, with two investigators (Nancy Rivera and Elana A. Feldman) reviewing the data independently and then comparing line by line until consensus was achieved. A third investigator (Rebecca Blankenburg) helped reconcile any conflicts. Frequency counts were determined by the number of times a theme was mentioned by any of the participants.

Institutional Review Board
The postworkshop evaluation form was reviewed by the Institutional Review Board of Stanford University (Protocol Number 45076) and was determined not to qualify as human subjects research.

Results

This workshop was facilitated by eight presenters: two residents, one fellow, one staff member, and four faculty members. It was presented at three local academic conferences (2019 Stanford Diversity and Inclusion Forum, 2019 Stanford Medicine Radiology Diversity Fair, and 2019 Leadership Education in Advancing Diversity Seminar). The workshop was also accepted at one national conference, the Pediatric Academic Societies annual meeting; however, due to COVID-19 restrictions, it was not presented. There was a total of 92 participants, comprising residents, fellows, staff, faculty members, and program and department leadership.

Ninety-two percent of participants felt the workshop met its learning objectives, while 90% of participants felt the workshop was a valuable use of their time. Furthermore, 89% of participants felt the supplemental handouts were useful, and 90% of participants said they would apply information learned at the workshop in the future (Table 1).

Participants were asked to indicate two things that they would do as a result of the workshop. Strategies emerged on individual, peer, and institutional levels (Table 2). On an individual level, seeking mentorship (15% of responses), practicing self-compassion (21% of responses), allowing for vulnerability (15% of responses), and having physical reminders of personal accomplishments (13% of responses) were frequent strategies that emerged as a result of the workshop. On a peer level, the majority of participants indicated they would attempt to celebrate the successes of their peers (22% of responses) and to alert others to their own IS (23% of responses). On an institutional level, participants wanted to increase institutional education on IS (29% of responses).

Table 1. Participant Ratings of the Workshop Experience

Item	Agree or Strongly Agree: No. (%)
Workshop met objectives	85/92 (92)
Workshop was a valuable use of my time	83/92 (90)
Handouts include useful resources	72/82 (88)
I will apply information learned today to develop a workshop in the future	83/92 (90)

Participants were asked to indicate barriers to implementing what they had learned from the workshop. Themes emerged on individual, peer, and institutional levels (Table 2). Barriers on an individual level included ingrained thought processes (11% of responses) and self-doubt (16% of responses). On a peer level, lack of support (22% of responses) and lack of openness with others (11% of responses) were identified as barriers. Lastly, institutional barriers included difficulty to change the culture within departments (22% of responses) as well as daily workload pressures (13% of responses).

Of the various workshop components, the small-group discussions were the most preferred among the participants.

Discussion

We developed and implemented a workshop to provide a framework for health care professionals to understand, acknowledge, and confront IS in the health care workforce. The workshop was well received by participants, and their feedback indicated that this is an important area that is not discussed or taught regularly. In presenting this workshop, we learned several lessons. The most important lesson was that familiarity with the concept of IS and experiences of IS varied widely. Some groups wanted an introduction to the concept of IS, while other groups wanted to explore mitigating the effects of IS more deeply. Because the most helpful part of the workshop for many people was small-group discussion of strategies to mitigate IS, the workshop has been designed to incorporate flexibility and group participation.

We encountered a few barriers when running the workshop. The first was how to successfully lead an interprofessional group through a joint workshop. We initially focused on separating groups by professional role/training level and using scenarios that specifically applied to each group so that the groups could teach each other about their own experiences of IS. However, we found that participants much preferred to be in randomized groups and appreciated more generalized scenarios. Participants

Copyright © 2021 Rivera et al. This is an open-access publication distributed under the terms of the Creative Commons Attribution license.

Table 2. Actions and Barriers Identified by Workshop Participants to Combat Imposter Syndrome

Actions/Barriers by Level	% of Responses by Participants	Representative Quote
Actions participants identified they would take following the workshop		
Individual level		
Seek mentorship	15	"I will continue to seek mentors both in/outside my area of interest."
Self-compassion	21	"Change the ticker tape running in my mind, as the participant recommended replacing some self-doubt phrases with more kind and compassionate phrases."
Physical reminder of accomplishments and strategies	13	"Put together a box filled with positive evaluations/letters for days I'm feeling like an imposter."
Allow for vulnerability	15	"Continue with my vulnerability and share my Imposter Syndrome moments."
Peer level		
Celebrate the successes of peers	22	"Lift others up—use our forum to give voice/platform to people who might not be seen as 'peers'."
Help others recognize imposter syndrome in themselves	23	"Be more intentional in letting others know we all experience imposter syndrome."
Institutional level		
Institutional education on imposter syndrome	29	"Remind program of need to be aware of Imposter Syndrome—to incorporate countermeasures."
Barriers participants identified that would impact doing the above actions		
Individual level		
Ingrained thought process	11	"For myself my own entrenched habits of thought."
Self-doubt	16	"I am my own enemy."
Peer level		
Lack of support from others	22	"Lack of support. Lots of my friends didn't attend, how do I explain what support I need."
Lack of openness with peers	11	"Lack of talk between people in leadership & juniors sharing experiences."
Institutional level		
Difficulty to change culture within department and institution	22	"My voice may matter in this room, but I have no avenue to institutional input that feels viable."
Time and workload pressures	13	"In times of high stress/disappointment, easy to slip back into [imposter syndrome]."

preferred discussing IS in randomized groups as this would help impact change across departments and the institution on a greater level. We also found that some groups that initially had no experience with IS were more reluctant to engage at first. This situation was improved by an initial didactic session and techniques like the word cloud, which allowed anonymous responses.

This workshop has been designed to be generalizable to many settings; however, it requires flexibility depending on a group's previous knowledge of the subject. The multiple scenarios allow workshop facilitators to pick what is most relevant for their group. The modalities (PowerPoint slides, cases, small-group discussion, and large-group discussion) have been designed to be easy to implement in many settings.

Limitations

Our workshop has several limitations. Participants might have been affected by social desirability bias, although we attempted to reduce this by using anonymous evaluations. Without demographic information, we cannot determine who would benefit from the workshop. Future studies can include a survey collecting demographic information separate from the workshop evaluation. We measured participants' intended behavior change, and future studies should explore their actual behavior change.

Conclusions

We successfully developed a workshop to help health care trainees, faculty, staff, and other members of the health care workforce discuss a framework for understanding IS as well as mitigating IS in their own professional contexts. The workshop empowers participants to recognize IS in themselves, their coworkers, and their mentees and can help foster a culture of support in professional advancement. As IS most affects those who have been historically underrepresented in medicine, this workshop and others like it can influence advancement at all levels.

Appendices

A. Facilitator Guide.docx

B. Workshop Handout.docx

C. Facilitator Lesson Plan.docx

Copyright © 2021 Rivera et al. This is an open-access publication distributed under the terms of the Creative Commons Attribution license.

D. PowerPoint Slides.pptx

E. Workshop Evaluation Form.docx

All appendices are peer reviewed as integral parts of the Original Publication.

Nancy Rivera, MD, MS: Resident, Department of Pediatrics, Stanford School of Medicine

Elana A. Feldman, MD: Clinical Assistant Professor, Department of Pediatrics, University of Washington School of Medicine

Dimitri A. Augustin, MD: Independent Practice

Wendy Caceres, MD: Clinical Assistant Professor, Department of Medicine, Stanford School of Medicine

Hayley A. Gans, MD: Clinical Professor, Department of Pediatrics, Stanford School of Medicine

Rebecca Blankenburg, MD, MPH: Clinical Professor, Department of Pediatrics, Stanford School of Medicine

Acknowledgments
With gratitude to Dr. Amanda Rigas, Taranjit Bains, and Stanford Leadership Education in Advancing Diversity (LEAD) Program for their contributions to the development, implementation, and mentorship of this workshop.

Disclosures
None to report.

Funding/Support
None to report.

Ethical Approval
The Stanford University Institutional Review Board approved this study.

References

1. Clance PR, Imes SA. The imposter phenomenon in high achieving women: dynamics and therapeutic intervention. *Psychotherapy (Chic).* 1978;15(3):241-247. https://doi.org/10.1037/h0086006
2. Sakulku J. The impostor phenomenon. *Int J Behav Sci*. 2011;6(1):75-97. https://doi.org/10.14456/ijbs.2011.6
3. Villwock JA, Sobin LB, Koester LA, Harris TM. Impostor syndrome and burnout among American medical students: a pilot study. *Int J Med Educ*. 2016;7:364-369. https://doi.org/10.5116/ijme.5801.eac4
4. Legassie J, Zibrowski EM, Goldszmidt MA. Measuring resident well-being: impostorism and burnout syndrome in residency. *J Gen Intern Med*. 2008;23(7):1090-1094. https://doi.org/10.1007/s11606-008-0536-x
5. LaDonna KA, Ginsburg S, Watling C. "Rising to the level of your incompetence": what physicians' self-assessment of their performance reveals about the imposter syndrome in medicine. *Acad Med*. 2018;93(5):763-768. https://doi.org/10.1097/ACM.0000000000002046
6. Holliday AM, Gheihman G, Cooper C, et al. High prevalence of imposterism among female Harvard medical and dental students. *J Gen Intern Med*. 2020;35(8):2499-2501. https://doi.org/10.1007/s11606-019-05441-5
7. Oriel K, Plane MB, Mundt M. Family medicine residents and the impostor phenomenon. *Fam Med*. 2004;36(4):248-252.
8. Bynum WE, Uijtdehaage S, Artino AR, Fox JW. The psychology of shame: a resilience seminar for medical students. *MedEdPORTAL*. 2020;16:11052. https://doi.org/10.15766/mep_2374-8265.11052
9. Bravata DM, Watts SA, Keefer AL, et al. Prevalence, predictors, and treatment of impostor syndrome: a systematic review. *J Gen Intern Med*. 2020;35(4):1252-1275. https://doi.org/10.1007/s11606-019-05364-1
10. Chrousos GP, Mentis AFA. Imposter syndrome threatens diversity. *Science*. 2020;367(6479):749-750. https://doi.org/10.1126/science.aba8039
11. Baumann N, Faulk C, Vanderlan J, Chen J, Bhayani RK. Small-group discussion sessions on imposter syndrome. *MedEdPORTAL*. 2020;16:11004. https://doi.org/10.15766/mep_2374-8265.11004
12. Ramsey JL, Spencer AL. Interns and imposter syndrome: proactively addressing resilience. *Med Educ*. 2019;53(5):504-505. https://doi.org/10.1111/medu.13852
13. Haney TS, Birkholz L, Rutledge C. A workshop for addressing the impact of the imposter syndrome on clinical nurse specialists. *Clin Nurse Spec*. 2018;32(4):189-194. https://doi.org/10.1097/NUR.0000000000000386
14. Metz CJ, Ballard E, Metz MJ. The stress of success: an online module to help first-year dental students cope with the impostor phenomenon. *J Dent Educ*. 2020;84(9):1016-1024. https://doi.org/10.1002/jdd.12181
15. Thomas PA, Kern DE, Hughes MT, Chen BY, eds. *Curriculum Development for Medical Education: A Six-Step Approach.* 3rd ed. Johns Hopkins University Press; 2016.
16. Anderson LW, Krathwohl DR, eds. *A Taxonomy for Learning, Teaching, and Assessing: A Revision of Bloom's Taxonomy of Educational Objectives.* Longman Press; 2000.
17. Cox E. What is imposter syndrome and how can you combat it? TED. August 2018. https://www.ted.com/talks/elizabeth_cox_what_is_imposter_syndrome_and_how_can_you_combat_it?utm_campaign=tedspread&utm_medium=referral&utm_source=tedcomshare

Received: September 8, 2020
Accepted: April 4, 2021
Published: July 6, 2021

Copyright © 2021 Rivera et al. This is an open-access publication distributed under the terms of the Creative Commons Attribution license.
